# Influence of sulfide on diazotrophic growth of the methanogen *Methanococcus maripaludis* and its implications for the origin of nitrogenase

**DOI:** 10.1038/s42003-023-05163-9

**Published:** 2023-07-31

**Authors:** Devon Payne, Rachel L. Spietz, Dennis L. Newell, Paul Dijkstra, Eric S. Boyd

**Affiliations:** 1 Department of Microbiology and Cell Biology, Montana State University, Bozeman, MT 59717 USA; 2 Department of Geosciences, Utah State University, Logan, UT 84322 USA; 3 Center for Ecosystem Science and Society and Department of Biological Sciences, Northern Arizona University, Flagstaff, AZ 86011 USA

**Keywords:** Water microbiology, Element cycles, Microbial ecology

## Abstract

Methanogens inhabit euxinic (sulfide-rich) or ferruginous (iron-rich) environments that promote the precipitation of transition metals as metal sulfides, such as pyrite, reducing metal or sulfur availability. Such environments have been common throughout Earth’s history raising the question as to how anaerobes obtain(ed) these elements for the synthesis of enzyme cofactors. Here, we show a methanogen can synthesize molybdenum nitrogenase metallocofactors from pyrite as the source of iron and sulfur, enabling nitrogen fixation. Pyrite-grown, nitrogen-fixing cells grow faster and require 25-fold less molybdenum than cells grown under euxinic conditions. Growth yields are 3 to 8 times higher in cultures grown under ferruginous relative to euxinic conditions. Physiological, transcriptomic, and geochemical data indicate these observations are due to sulfide-promoted metal limitation, in particular molybdenum. These findings suggest that molybdenum nitrogenase may have originated in a ferruginous environment that titrated sulfide to form pyrite, facilitating the availability of sufficient iron, sulfur, and molybdenum for cofactor biosynthesis.

## Abstract (web summary)

Iron, sulfur, and molybdenum availability are shown to affect growth and metabolic characteristics of *Methanococcus maripaludis* in such a way that suggests molybdenum nitrogenase may have originated in a ferruginous environment.

## Introduction

Nitrogen (N) is essential for the synthesis of nucleic and amino acids and other key biomolecules in all forms of life. Earth’s largest reservoir of N is dinitrogen (N_2_) gas in the atmosphere; however, it is not bioavailable and must be fixed to nitrate (NO_3_^-^) or ammonia (NH_3_) prior to its assimilation. As such, the availability of fixed N often limits the productivity of ecosystems^1^. On early Earth, fixed N is thought to have been supplied by abiotic processes such as lightning-based oxidation of atmospheric N_2_ or mineral reduction of N_2_^2,3^. However, fixed N from these sources would have been minimal and finite, and together these features are thought to have limited ecosystem productivity during this time^1^. Today, approximately 50% of all fixed N is generated through the biological process of N_2_ fixation^1,4^, whereby N_2_ is reduced to NH_3_ by the enzyme nitrogenase (diazotrophy) with the remaining fixed N largely generated through the industrial Haber-Bosch process.

Three different forms of nitrogenase have been described to date and these are differentiated by the (hetero)metals comprising the active site of each enzyme complex^5^. This includes molybdenum (Mo), vanadium (V), and iron (Fe)-only forms of nitrogenase^6,7^. Mo-nitrogenase (Nif) is taxonomically the most widely distributed and oldest form of nitrogenase^8,9^ and, at a minimum, consists of the structural proteins NifHDK and maturase proteins, NifB(E)N^10^. The active site metallocluster of Nif comprises a six iron (Fe) and nine sulfur (S) atom core with Fe symmetrically coordinating a central carbon atom; the metallocluster is capped by Fe and Mo atoms [7Fe-Mo-9S] (termed the FeMo-cofactor^10,11^). In addition to FeMo-cofactor(s), Nif requires the complex P-cluster that consists of eight Fe atoms and seven S atoms [8Fe-7S]^12^. A FeMo-cofactor is housed within each NifD structural protein, whereas the P-cluster is found at the interface of each NifD and NifK, ultimately forming the heterotetramer, NifD_2_K_2_^13^. Dinitrogenase reductase, NifH, modulates the ATP-dependent transfer of electrons to NifD_2_K_2_ and it harbors an additional [4Fe-4S] cluster for each of two NifH subunits^14,15^. Thus, cells performing N_2_-fixation via Nif have a high demand for Fe, S, Mo, ATP, and reducing equivalents.

Phylogenetic analyses of a concatenation of NifHDK proteins indicate that the earliest evolving lineages of Nif are found in hydrogenotrophic methanogens^6–9,16,17^. These observations are corroborated by other data indicating NifHDK evolved from a series of duplications of genes encoding an ancestor of CfbCD^8^, proteins that are required to synthesize cofactor F_430_^18,19^. That F_430_ (and CfbCD encoding genes) are exclusively found in archaeal methanogens (and archaeal alkanotrophs)^20^ is further evidence indicating an origin for Nif among ancestors of these Archaea. These data have been used to suggest an origin for Nif among an ancestor of anaerobic methanogens during the mid-Paleoproterozoic ~1.8–2.1 billion years ago (Ga)^6,9,17^, although isotopic data of organic matter preserved in shales dated to >3 Ga suggest an even earlier origin^21,22^. Regardless of the actual date of its origin, nitrogenase is interpreted to be an ancient enzyme that originated in an anoxic environment on early Earth. An anoxic origin for this enzyme is consistent with the oxygen-sensitivity of the metal clusters required for Nif function^23^. Nif then diversified among anaerobes and only late in its evolutionary history was it acquired via horizontal gene transfer among organisms capable of integrating oxygen (O_2_) into their energy metabolism or capable of producing O_2_ in the case of Cyanobacteria. Expanded biological productivity associated with the proliferation of Cyanobacteria and O_2_ production would have increased demand for existing abiotic pools of fixed N^24^, which may have been the selective pressure to evolve a biological mechanism to reduce atmospheric N_2_ and relieve N limitation^7,25^.

Ferruginous conditions (anoxic and ferrous iron (Fe(II))-rich) likely dominated anoxic environments during the Archean (>2.4 Ga)^26,27^. This is due to the circulation of hydrothermal fluids through iron-rich mid-ocean basalts, which led to input of a greater amount of Fe(II) into anoxic ocean basin waters than sulfide (HS-)^28^. In the absence of oxygen, the excess Fe(II) would have been stable, and free Fe(II) concentrations are estimated to have ranged from 0.05 to 0.5 mM^29^. However, the proliferation of Cyanobacteria and the production of O_2_ during the late Archean drove the oxidative weathering of continental sulfide minerals that increased the flux of sulfate into oceans. When combined with increased productivity near ocean margins^30,31^, this would have stimulated heterotrophic sulfate reduction and HS^-^ production. In turn, this led to stratified coastal oceans that were oxygenated at the surface and were euxinic (anoxic and HS^-^-rich) at depth^30–32^. In contrast, deeper ocean waters and those more distal from continental margins remained ferruginous^26^, due to lower productivity in the overlying water column, decreased availability of sulfate and subsequent heterotrophic sulfate reduction, and hydrothermal input of Fe^26,33,34^. HS^-^ has a high affinity for Fe(II), resulting in the formation of low-solubility iron-sulfide minerals, including pyrite (FeS_2_)^35–37^. As such, in euxinic environments, concentrations of HS^-^ exceed that of Fe(II), resulting in the titration and precipitation of Fe(II) as FeS_2_. Under these conditions, excess HS^-^ is also available to complex with other thiophilic metals (e.g., Mo, Co, Ni), potentially rendering them less bioavailable^33,34,38^.

How might N_2_-fixing methanogens have met their concurrent demands for Fe, S, and Mo for the synthesis of Nif cofactors during the Paleoproterozoic or even earlier, when one or more of these elements were likely to be of limited availability due to the tendency for metal sulfide formation? Potential clues come from recent studies of contemporary methanogens, specifically *Methanococcus voltae* and *Methanosarcina barkeri*, that reveal their ability to reductively dissolve FeS_2_ and utilize dissolution products to meet Fe and S nutritional demands^39–41^. *M. voltae* and *M. barkeri* grown with FeS_2_ had similar growth rates and cell yields when compared to traditional sources of Fe and S used to grow methanogens (Fe(II) and HS^-^ and/or cysteine)^39,40^. However, these experiments were conducted on non-N_2_-fixing methanogen cells, which would be expected to have a lower demand for Fe, S, and Mo than those actively fixing N_2_, in particular those growing with Mo-nitrogenase. Such observations point to ferruginous conditions as being possibly more favorable than euxinic conditions for the origin and early proliferation of Nif.

Here, we sought to evaluate the effect of euxinic and ferruginous conditions on the growth and activity of the methanogen, *Methanococcus maripaludis* S2 (MmS2), to provide new insights into the environment type most conducive for the origin of Nif. Cells were first grown with Fe(II) and HS^-^ or FeS_2_ as a primary Fe and S source under N_2_-fixing or NH_3_-amended conditions, to establish whether FeS_2_ could serve as a Fe and S source for biosynthesis of nitrogenase and to determine whether HS^-^ drives Mo limitation. Cultivation assays were then focused on FeS_2_-grown cells, since this condition allows for excess Fe(II) or HS^-^ to be included in the cultivation medium thereby permitting an evaluation of the effect of ferruginous or euxinic conditions, respectively, on metal availability and N_2_ fixation activity. Nif is the only nitrogenase encoded in MmS2^42^, mitigating the confounding variable associated with cells switching to more recently evolved and alternative forms of nitrogenase (i.e., Fe-nitrogenase Anf; V-nitrogenase, Vnf^9^) if, or when, Mo becomes limiting^5,43^. Further, the Nif encoded by MmS2 belongs to the earliest evolving lineage of nitrogenase^6,8,9,16^. To place additional Mo demands on MmS2, cells were grown with formate, requiring the expression of the molybdopterin-containing formate dehydrogenase^44,45^ and Mo- or tungsten (W)-dependent formylmethanofuran dehydrogenase (Fmd and Fwd, respectively)^46,47^. Results are discussed as they relate to the potential environmental condition (i.e., euxinic or ferruginous) that would have allowed the O_2_-sensitive Nif enzyme to evolve and function in a methanogen, and ultimately other anaerobes, in the anoxic habitats of early and present-day Earth.

## Results and discussion

### Nitrogen fixation and Fe/S source affect *Methanococcus maripaludis* S2 (MmS2) Growth and Activity

**Fig. 1:**
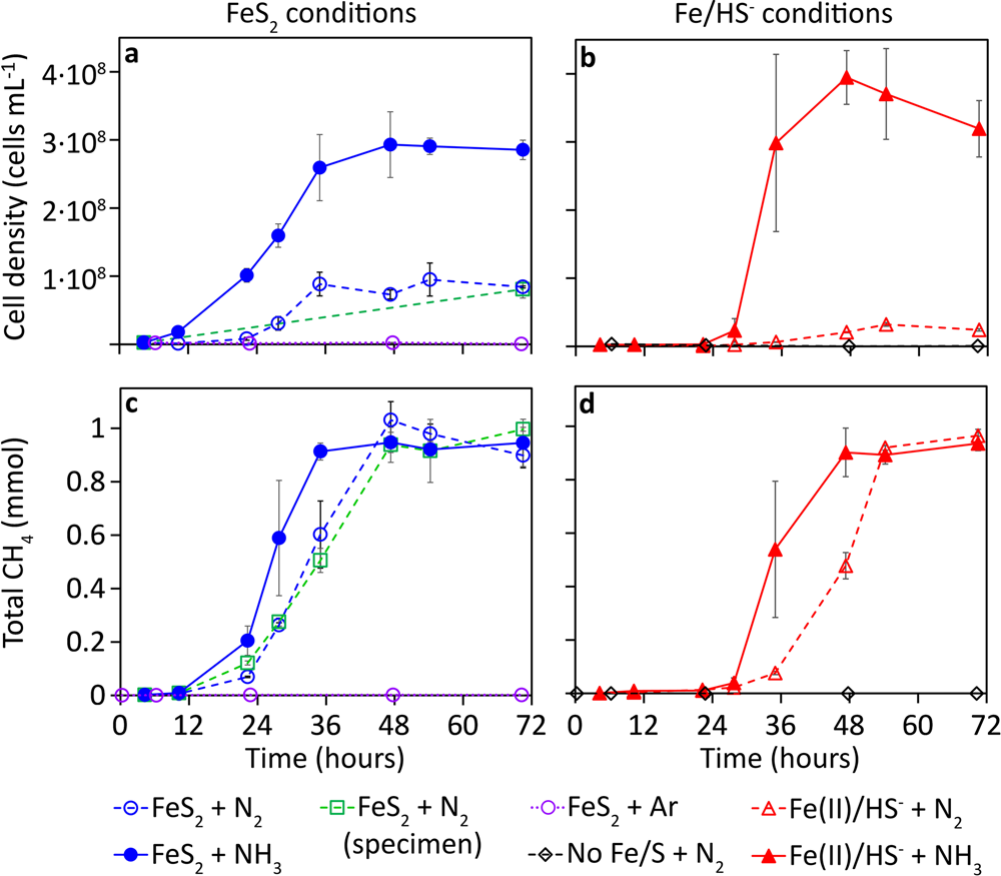
Growth of *Methanococcus maripaludis* with formate as methanogenesis substrate under nitrogen-fixing and ammonia-amended growth conditions with pyrite or ferrous iron and sulfide as the sole provided iron and sulfur source. Growth and activity were monitored by quantifying cell density (**a**, **b**) and methane production (**c**, **d**) over time, respectively. Panels **a** and **b** and panels **c** and **d** are plotted using the same *y*-axes, respectively. The data presented are the mean and standard deviation of three biological replicates per condition. ammonia NH_3_, argon Ar, dinitrogen N_2_, ferrous iron Fe(II), methane CH_4_, pyrite FeS_2_, sulfide HS^-^.

MmS2 grew in both N_2_-fixing and non-N_2_-fixing (NH_3_-amended) growth conditions when synthetic nanoparticulate FeS_2_ or Fe(II)/HS^-^ were provided as the sole Fe/S sources and with formate as the electron donor and methanogenesis substrate (Fig. 1a–d). Growth did not occur in cultures that were not provided with an Fe/S source, regardless of whether cells were provided with N_2_ or NH_3_. Likewise, cultures grown in the absence of NH_3_ and under a headspace of argon (Ar) did not grow, regardless of the Fe/S source provided (Fig. 1a, c). Importantly, MmS2 was also able to grow under N_2_-fixing conditions with specimen (63–150 µm grain size) FeS_2_, indicating that a natural (not laboratory-synthesized) form of FeS_2_ provides Fe and S during N_2_ fixation (Fig. 1a, b). Cell counts and CH_4_ measurements were performed infrequently in cultures with specimen FeS_2_ since preliminary experiments showed they were sensitive to mechanical disturbance. This may be related to the different geometries of the synthetic nanoparticulate FeS_2_ that is composed of small framboids with high surface area compared to the large and flat facies of the mostly cubic specimen FeS_2_, which could affect the ability for cells to attach (Supplementary Fig. 1). Nonetheless, these data indicate that both synthetic and natural forms of FeS_2_ can serve as the sole Fe/S source in N_2_-fixing MmS2 cells.

The total number of cells produced in the NH_3_-amended cultures was higher relative to N_2_-fixing cultures, regardless of the source of Fe/S provided (Fig. 1a, b). Despite differences in final cell densities, cultures grown under all conditions (except for negative controls) produced similar amounts of CH_4_ by 54 h incubation (Fig. 1c, d). As such, the cell yield (cells per mmol CH_4_) in N_2_-fixing cultures was significantly (*p* < 0.01) lower than in NH_3_-amended cultures (Supplementary Fig. 2). This finding is consistent with a nearly three-fold decrease in the cell yield of the methanogen, *Methanothermobacter thermolithotrophicus*, when grown under N_2_-fixing conditions relative to NH_3_-amended conditions^48^. The source of Fe and S provided to MmS2 cultures also had a marked effect on the rate of cell production (Supplementary Table 1) and the total number of cells produced for N_2_-fixing conditions but had minimal effect on NH_3_-amended cultures (Fig. 1a, b). Specifically, N_2_-fixing cultures provided with FeS_2_ had a nearly three-fold higher (*p* < 0.01) cell yield than those provided with Fe(II)/HS^-^ (Supplementary Fig. 2). In contrast to past reports that showed NH_3_-amended cultures of *M. voltae* and *M. barkeri* (both strains MS and Fusaro) achieved similar cell yields when provided with FeS_2_ or Fe(II)/HS^-^^40,41^, FeS_2_-grown MmS2 had a yield that was 29% lower than Fe(II)/HS^-^-grown cells provided with NH_3_. However, the reduction in cell yield was even more dramatic when N_2_-fixing MmS2 cells were compared, with those provided with Fe(II)/HS^-^ exhibiting a 92% reduction in yield relative to those provided NH_3_. This demonstrates the energetic burden N_2_ fixation places on MmS2 cells and the dependency of this on the Fe/S source provided.

### Nitrogen fixation and Fe/S source affect MmS2 cell size

To better understand differences in the growth kinetics and yields observed in cultures of MmS2 grown under N_2_-fixing or NH_3_-amended conditions with FeS_2_ or Fe(II)/HS^-^ as the sole Fe/S source, the size of cells during the log phase in each growth condition was determined using field-emission scanning electron microscopy (FEM). The average size of MmS2 cells was found to be significantly different (*p* < 0.05) for each growth condition, where NH_3_-amended cells grown with Fe(II)/HS^-^ were the largest followed by N_2_-fixing cells grown with Fe(II)/HS^-^. The NH_3_-amended cells grown with FeS_2_ were smaller yet and N_2_-fixing cells grown with FeS_2_ were the smallest in size among all treatments (Supplementary Table 1).

Cell size is suggested to be positively correlated with cell growth rate (cell divisions hr^−1^) and/or nutrient availability, although the regulatory mechanisms for cell size are poorly understood across all domains of life^49,50^. The “growth law” suggests cell size is positively correlated with growth rate^50^; however, this fails to explain the patterns observed here. Diazotrophic MmS2 cells grown on Fe(II)/HS^-^ had the second largest size but the lowest of the observed growth rates across all four conditions (Supplementary Table 1). Rather, these results indicate that growth on FeS_2_ imparts a greater reduction in cell size than N_2_ fixation, and that these effects are semi-additive. This is consistent with recent data that suggest that cell size is more of a function of nutrient availability than growth rate alone^49^, with nutrient-limited conditions leading to smaller cell sizes possibly due to the physiological advantage of increased surface area to volume ratios for nutrient transport across the membrane. For example, the diazotrophic marine cyanobacterium, *Crocosphaera watsonii*, was demonstrated to undergo a ~2.2-fold reduction in cell volume in response to Fe limitation^51^. Similarly, *M. voltae* cells showed a ~3.2-fold reduction in cell volume when grown on FeS_2_ relative to Fe(II)/HS^-^^39^. Proteomic analysis of *M. voltae* cells revealed an up-expression of iron uptake proteins (Feo) when grown with FeS_2_, indicating cells may incorrectly sense Fe(II) limitation due to assimilation of Fe(II) complexed with sulfide (FeS_(aq)_), despite FeS_2_-grown cells having higher Fe content^39^. In comparison, N limitation has also been shown to lead to decreased cell size, with ~1.5-to-2.5-fold reduction in cell volume in heterotrophic bacterioplankton isolates grown under N-limiting conditions^52,53^. The semi-additive effect of Fe, S, and N sources and availability on MmS2 cell size and growth rate suggest that MmS2 cells are responding to real or perceived limitations of one or more of these elements.

### Nitrogen fixation and Fe/S source affect C and N isotope compositions in MmS2 biomass and CH_4_

The isotopic composition of C in biomass and CH_4_ has been shown to vary with growth rate, substrate availability, and environmental conditions in a variety of methanogen cultures^54–58^. To further evaluate the effect of N availability and Fe and S source on the phenotype of MmS2, and to confirm N_2_ fixation activity, stable isotopes of C (δ^13^C) and N (δ^15^N) in CH_4_ and/or biomass were examined in cultures grown under N_2_-fixing or NH_3_-amended conditions with either FeS_2_ or Fe(II)/HS^-^ provided as the sole Fe/S source.

**Fig. 2:**
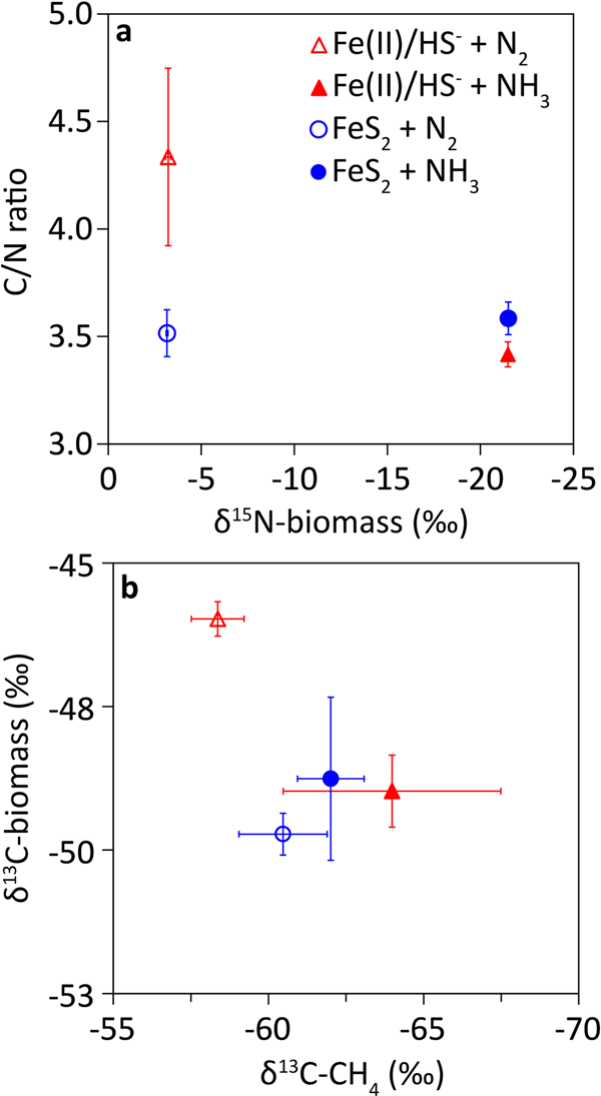
Isotopic composition of *Methanococcus maripaludis* biomass and methane when grown with formate as methanogenesis substrate under nitrogen-fixing or ammonia-amended conditions with pyrite or ferrous iron and sulfide as the sole provided iron and sulfur source. Mid-log-phase cells (biomass) were analyzed for their carbon-to-nitrogen (*C*/*N*) mass ratios and δ^15^N values (**a**). Biomass and methane were also analyzed for δ^13^C values (**b**). The legend in a is the same for **b**. The data presented are the mean and standard deviation of three replicate cultures for all conditions. Isotope results are presented in the delta notation as per mille (‰) values vs. Air (δ^15^N) or VPDB (δ^13^C). See Supplementary Data 1 for additional data. Ammonia NH_3,_ dinitrogen N_2,_ ferrous iron Fe(II), methane CH_4,_ pyrite FeS_2,_ sulfide HS^-^ .

MmS2 cell biomass had distinctly different δ^15^N values when grown under N_2_-fixing vs. NH_3_-amended conditions (Fig. 2a). Cells grown under N_2_-fixing conditions had similar δ^15^N values of −3.22 ± 0.14 and −3.16 ± 0.06‰ when provided with Fe(II)/HS^-^ and FeS_2_, respectively. These slight differences in δ^15^N values are not statistically significant (*p* = 0.55) and are similar to δ^15^N values of −4‰ previously reported for N_2_-fixing *Methanocaldococcus* and *Methanothermococcus* strains isolated from hydrothermal vents^59^. NH_3_-amended MmS2 cells grown with Fe(II)/HS^-^ and FeS_2_ had nearly identical δ^15^N values of −21.63 ± 0.23 and −21.61 ± 0.47‰, respectively. Assimilation of NH_3_ by microorganisms such as *E. coli* is known to produce biomass with δ^15^N values ranging from −16 to −21‰ under NH_3_ replete conditions^60^. The NH_4_Cl salt used to cultivate MmS2 had a δ^15^N value of -3.06‰ (Fig. 2a); as such, the fractionation of δ^15^N in biomass would be closer to ~ −18.6‰ and therefore is within the range of expected values for cells assimilating NH_3_.

The δ^13^C values for MmS2 biomass were similar for all growth conditions tested (−48 to −50‰), with the exception of biomass grown under N_2_-fixing conditions with Fe(II)/HS^-^ (−46‰; Fig. 2b). Previous studies have interpreted decreased fractionation of biomass C isotopes in methanogens to indicate lower turnover of C substrates^54^, and this is consistent with the slow growth rate and low cell densities observed in the N_2_-fixing condition with Fe(II)/HS^-^. Nonetheless, all biomass δ^13^C values are within the range of values of biomass (range −30 to −49‰) previously determined for a variety of methanogens when grown with various electron donors/carbon sources across a range of cultivation conditions^54^. The δ^13^C values of CH_4_ were overall similar (−58 to −64‰) with the exception of N_2_-fixing cells provided with Fe(II)/HS^-^, which was heavier (−58‰) but not significantly different (*p* = 0.07) than that of N_2_-fixing cells grown with FeS_2_ (−60‰) (Fig. 2b). Like the δ^13^C biomass, the δ^13^C of CH_4_ is within the range of values (−50 to −100‰) measured for methanogen cultures when grown with a variety of electron donors/methanogenesis substrates across a range of cultivation conditions^54,55,61^. Together, these results suggest that N_2_ fixation impacts the carbon and energy metabolism of MmS2, and this effect is greater when N_2_-fixing cells are provided with Fe(II)/HS^-^ when compared to FeS_2_. This could be due to competition for molybdate between formate dehydrogenases, nitrogenases, and potentially formylmethanofuran dehydrogenases, which may be amplified by HS^-^-promoted decreases in the availability of Mo (discussed more below). This would be expected to decrease the rate of formate oxidation and CO_2_ production, allowing for the passive exchange of intracellular and extracellular CO_2_ to have a larger influence on the isotopic composition of CH_4_.

In addition to investigating C and N isotopes, the masses of C and N and *C*/*N* ratios were determined for biomass samples (Fig. 2a). The *C*/*N* ratios were similar across all conditions (range of 3.4 to 3.6), except N_2_-fixing MmS2 cells grown on Fe(II)/HS^-^ that had a *C*/*N* ratio of 4.3. Previous studies have shown that *C*/*N* ratios in cultures of heterotrophic marine bacterioplankton increase by ~40% when limited to fixed N^52^. Furthermore, the *C*/*N* ratio of N_2_-fixing *Nostoc* cultures increased by 80% under MoO_4_^2-^ limitation^62^. The elevated *C*/*N* ratio of N_2_-fixing MmS2 cells provided with Fe(II)/HS^-^ (Fig. 2a), when considered in light of the low cell yields in this condition (Supplementary Fig. 2), points to N and/or MoO_4_^2-^ limitation in these cells. Importantly, differences in the autotrophic pathways used by methanogens (Wood-Ljungdahl) and oxygenic phototrophs (Calvin Cycle) may influence the partitioning of carbon into biomass or CH_4_ and may influence the *C*/*N* ratios in biomass that are being compared here.

### Nitrogen fixation and Fe/S source affect MmS2 gene expression

**Fig. 3:**
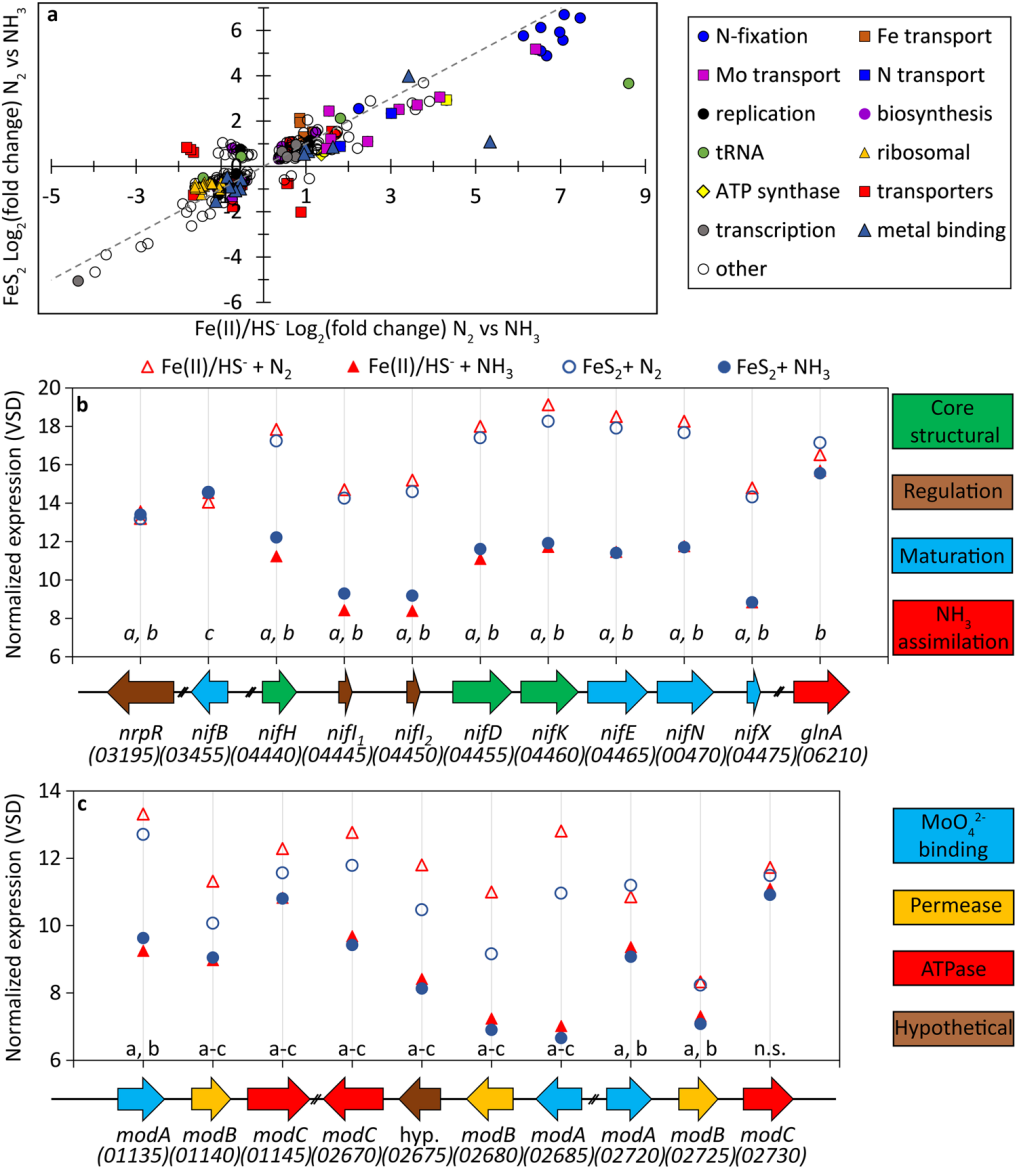
Differential transcription of genes in *Methanococcus maripaludis* cultures grown under nitrogen-fixing or ammonia-amended conditions with pyrite or ferrous iron and sulfide as the sole provided iron and sulfur source. **a** Transcripts that were significantly differentially expressed *(p* < 0.05, Wald test) for nitrogen-fixing relative to ammonia-amended cells when grown on ferrous iron and sulfide (*x*-axis) or pyrite (*y*-axis) shown on a log_2_-fold-change scale. Positive values indicate higher expression under nitrogen-fixing conditions, and negative values indicate higher expression under ammonia-amended conditions. A gray dashed 1:1 line is provided to ease comparisons based on iron and sulfur source. Mean transcript expression is shown for select genes related to nitrogen fixation (**b**) and molybdenum transport (**c**) that are organized in the order they appear in the genome. Each gene is represented by a color-coded point (**a**) or arrow (**b**, **c**) with locus tags designated in parentheses (“MMP_RS” has been truncated from the locus designation to conserve space). Genes are colored according to function in the legend to the right of each panel. Each point represents the mean normalized expression of three culture replicates per growth condition. Statistically significant (*p* < 0.05, Wald test) differences in expression for nitrogen-fixing vs. ammonia-amended conditions with ferrous iron and sulfide (**a**) or pyrite (**b**) or between nitrogen-fixing conditions grown with either iron and sulfur source (**c**) are shown above each gene for panels **b** and **c**. ferrous iron Fe(II), pyrite FeS_2_, sulfide HS^-^, dinitrogen N_2_, ammonia NH_3,_ no significance, n.s.

Shotgun transcriptomics was used to generate gene expression profiles from log-phase cultures of N_2_-fixing and NH_3_-amended cells provided with FeS_2_ or Fe(II)/HS^-^ as the sole Fe/S source (see Supplementary Table 2 for growth data). Differences in gene expression were then used to begin to identify the metabolic/physiological processes that were impacted by the N, Fe, and S sources that were provided. Overall, 1737 unique transcripts were detected among the 1742 protein-coding genes encoded by MmS2 (99.7% of genes had detectable transcripts). Principal component analysis of gene expression profiles revealed that samples clustered according to cultivation conditions (Supplementary Fig. 3). A variety of cellular processes (215 genes) were significantly (*p* < 0.05) differentially regulated between N_2_-fixing and NH_3_-amended cells grown with either Fe(II)/HS^-^ or FeS_2_ (Fig. 3a). The majority of the genes differentially expressed between N_2_-fixing and NH_3_-amended cells had similar log_2_-fold-change values for either Fe(II)/HS^-^ or FeS_2_ conditions (i.e., these genes fall on a 1:1 line in Fig. 3a), further emphasizing that N_2_ fixation induces a significant physiological response regardless of the Fe and S source. Of these genes, those involved in N_2_ fixation, molybdenum and iron transport, and metal binding were significantly upregulated under N_2_-fixing conditions regardless of Fe and S source. Conversely, ribosomal genes, genes involved in cellular biosynthesis (e.g., acetyl-CoA synthase), and genome replication (e.g., DNA polymerase) were repressed under N_2_-fixing conditions. These results are consistent with decreased growth rates and yields observed for N_2_-fixing cells when compared to NH_3_-amended cells, regardless of the Fe and S source provided (Fig. 1).

Core *nif* genes are encoded in a single operon in MmS2^63^ that is transcriptionally regulated by the repressor protein NrpR and 2-oxoglutarate, a signal of N limitation^64–66^. The *nif* transcriptional regulator, NrpR, was not differentially transcribed based on the N source, which is consistent with previous reports that the activity of this repressor is independent of its levels in the cell (Fig. 3b)^67^. Genes encoding the core structural components of Nif (*nifHDK*), maturases (*nifENX*), and regulatory proteins (*nifI*_*1*_, *nifI*_*2*_) were upregulated in N_2_-fixing MmS2 cells relative to NH_3_-amended cells and patterns of their expression were generally similar regardless of whether cells were provided with Fe(II)/HS^-^ or FeS_2_ (Fig. 3b). A single *nif* gene, *nifB*, falls outside of the *nif* operon and was found to be significantly differentially expressed between N_2_-fixing conditions, with Fe(II)/HS^-^-grown cells having slightly lower levels of expression than FeS_2_-grown cells. NifB is responsible for the assembly of the FeMo-cofactor precursor, NifB-co, which is then transferred to NifEN where final cofactor maturation takes place through the addition of Mo and homocitrate^68^. It is not clear why the expression of NifB was lower in N_2_-fixing conditions in Fe(II)/HS^-^-grown cells. In addition to *nif* genes, expression of glutamine synthase (*glnA*) was elevated in N_2_-fixing cells relative to NH_3_-amended cells, but this difference was only significant for FeS_2_-grown cells. GlnA is an important enzyme for NH_3_ assimilation in MmS2^64^, and the higher expression in N_2_-fixing FeS_2_-grown cells may have contributed to their lower *C*/*N* ratios. Collectively, the transcriptomic profiles demonstrate that MmS2 upregulates the expression of genes encoding Nif maturase, Nif structural, and NH_3_ assimilation (i.e., GlnA) proteins when fixing N_2_ and expression of these genes is generally similar between the Fe/S sources.

Nif is the only nitrogenase encoded by MmS2 and this enzyme is Mo-dependent^42^. In addition, MmS2 encodes a Mo-dependent formate dehydrogenase (Fdh)^44,45^ and both Mo- and W-dependent forms of formylmethanofuran dehydrogenase (Fmd and Fwd, respectively) that are required for methanogenesis with formate^46,47^. Expression of *fdh* was overall similar among the conditions, with only one subunit encoding gene (*fdhB*; MMP_RS00800) that was significantly upregulated in NH_3_-amended conditions relative to N_2_-fixing conditions when grown with Fe(II)/HS^-^ (Supplementary Table 3). Interestingly, transcription of *fwd* and *fmd* differed among growth conditions, with three out of seven *fwd* genes (MMP_RS06415-MMP_RS06425) being significantly upregulated under NH_3_-amended conditions relative to N_2_-fixing conditions in Fe(II)/HS^-^ grown cells; expression of *fwd* genes was similar in NH_3_-amended conditions relative to N_2_-fixing conditions in FeS_2_-grown cells. In the case of *fmd*, the expression of an entire cluster of five *fmd* genes (MMP_RS02690-MMP_RS02710) was significantly upregulated (log_2_ fold-change of ~1.5) in N_2_-fixing cells relative to NH_3_-amended conditions when grown with FeS_2_; aside from a single gene *fmdE* (MMP_RS02690), *fmd* transcripts did not differ significantly in N_2_-fixing cells relative to NH_3_-amended cells when grown with Fe(II)/HS^-^ (Supplementary Table 3). It is unclear why *fwd* was up-expressed under NH_3_-amended conditions with Fe(II)/HS^-^ but it could be due to the very high affinity of MoO_4_^2-^ for HS^-^ and its low solubility^69^. This, in turn, would increase the bioavailability of tungstate (WO_4_^2-^) relative to MoO_4_^2-^, that when combined with the higher growth yields in the NH_3_-amended conditions with Fe(II)/HS^-^, leads to up-expression of *fwd* transcripts. Similarly, the increased transcription of *fmd* genes under N_2_-fixing conditions relative to NH_3_-amended conditions on FeS_2_ could indicate Mo is more available in FeS_2_ growth conditions, with an expression of *fwd* transcripts being unaffected.

Members of both Archaea and Bacteria acquire Mo in the form of MoO_4_^2-^ using MoO_4_^2-^-specific ABC transporters called Mod^70^. Transcripts for three *mod*-related gene clusters encoded by MmS2 were compared in N_2_-fixing and NH_3_-amended cells grown with FeS_2_ or Fe(II)/HS^-^ (Fig. 3c). Expression of genes comprising all three *mod* gene clusters was upregulated in N_2_-fixing cells relative to NH_3_-amended cells regardless of Fe/S source. Furthermore, expression of genes comprising two of the three *mod* gene clusters (MMP_RS01135-MMP_RS01145 and MMP_RS02670-MMP_RS02685) and an annotated NifC-like transporter (MMP_RS08475) were significantly upregulated (*p* < 0.05) in N_2_-fixing cells grown with Fe(II)/HS^-^ relative to those grown with FeS_2_. These results indicate that N_2_ fixation increases the expression of putative MoO_4_^2-^ transporters, and this expression is higher in N_2_-fixing cells grown with Fe(II)/HS^-^ when compared to those grown with FeS_2_. Collectively, these data suggest that MoO_4_^2-^ transport is influenced by not only demands associated with Mo-dependent enzymes (Fdh, Fmd, Nif) but also by Fe/S source. More specifically, the results suggest Mo limitation in N_2_-fixing cells grown with Fe(II)/HS^-^, possibly due to HS^-^ complexing and/or reacting with Mo^38,71^.

### Abiotic reactions with HS^-^ influence the speciation of Mo

A previous study reported that HS^-^ at a concentration of >6 mM inhibits N_2_ fixation in stream sediments^72^. While this effect was attributed to the toxicity of HS^-^ in cells responsible for N_2_ fixation, it is plausible that the added HS^-^ influenced the availability of trace metals (e.g., Fe, Mo) required for N_2_-fixing cells^62,73–75^. A similar effect of HS^-^ on metal availability in N_2_-fixing MmS2 cultures may explain the lower cell yields and increased expression of Mod genes observed in Fe(II)/HS^-^-grown cells relative to those grown with FeS_2_. MoO_4_^2-^ is a thiophilic molecule that readily reacts with HS^-^ to form soluble thiomolybdate (MoO_4-n_S_n_^2-^) species that can ultimately be incorporated in sulfide minerals, such as FeS_2_^71,76^. Such thiolation reactions would be expected to occur under the sulfidic (2 mM) cultivation conditions typically used to culture methanogens^77^ and that were utilized herein for the Fe(II)/HS^-^-grown cultures. These euxinic cultivation conditions potentially limited the availability of MoO_4_^2-^ such that it did not meet cellular demands.

To begin to examine this hypothesis, the speciation of Mo was tracked in abiotic reactors containing anoxic, base salts medium without added NH_3_ or metals. Reactors were amended with various combinations of HS^-^ (2 mM), Fe(II) (25 µM), or FeS_2_ (2 mM) to mimic cultivation conditions used in experiments up to this point. To these, Mo was added as MoO_4_^2-^ and the concentration of MoO_4_^2-^ and its conversion to MoO_4-n_S_n_^2-^ were monitored via colorimetric assays^78^ and UV–Vis spectroscopy^71^, respectively. Experiments were performed with 10 µM of MoO_4_^2-^ (as opposed to 4 µM used to cultivate MmS2 herein) to increase signal sensitivity such that it was within the dynamic range of the assays that were used.

MoO_4_^2-^ concentrations remained at ~8–10 µM in reactors not amended with HS^-^ in the presence or absence of FeS_2_ (Supplementary Fig. 4a). As expected, the product of complete thiolation of MoO_4_^2-^, tetrathiomolybdate (MoS_4_^2-^), was not detected in these conditions throughout the course of the experiment (Supplementary Fig. 4b). In contrast, MoO_4_^2-^ concentrations decreased rapidly (within the first 12 h of incubation) when HS^-^ alone or HS^-^ and Fe(II) were added to reactors (Supplementary Fig. 4a). In reactors containing HS^-^, MoS_4_^2-^ was rapidly produced (within the first hour of incubation) and MoO_4_^2-^ was completely converted to MoS_4_^2-^ by 72 h incubation. In reactors containing both HS^-^ and Fe(II), the conversion of MoO_4_^2-^ to MoS_4_^2-^ was accelerated, and complete conversion occurred within the first 3 h of incubation (Supplementary Fig. 4b). UV–Vis spectroscopy revealed production of MoO_4–*n*_S_*n*_^2-^ intermediates in reactors amended with HS^-^ with or without Fe(II) (Supplementary Fig. 5). Ions of MoO_4_^2-^/thiomolybdate have a strong affinity for Fe, including Fe in iron sulfides^79,80^, and it has been shown that these ions readily react to form stable Fe-Mo-S cubane-like structures in sulfur-rich environments^80^. It is possible that the acceleration of MoO_4_^2-^ thiolation by Fe(II) in the experiments described here is similarly attributable to the formation of stable Fe-Mo-S cubane-like clusters. Regardless, these data indicate that MoO_4_^2-^ is readily available in cultures grown with FeS_2_, while the predominant forms of Mo in cultures grown with Fe(II)/HS^-^ are a mixture of MoS_4_^2-^ and MoO_4-n_S_n_^2-^. This could potentially explain poor growth and up-expression of *mod* genes in N_2_-fixing cultures provided with Fe(II)/HS^-^ relative to FeS_2_. It is unknown if N_2_-fixing MmS2 cells in the Fe(II)/HS^-^ conditions are using sub-micromolar levels of MoO_4_^2-^ that are below the detection limit (~1 µM) of the colorimetric assay used herein or if they can use MoO_4-n_S_n_^2-^ (albeit to a limited extent based on growth data) to meet their Mo nutritional demands.

### HS^-^ influences the bioavailability of MoO_4_^2-^ and MmS2 growth kinetics

**Fig. 4:**
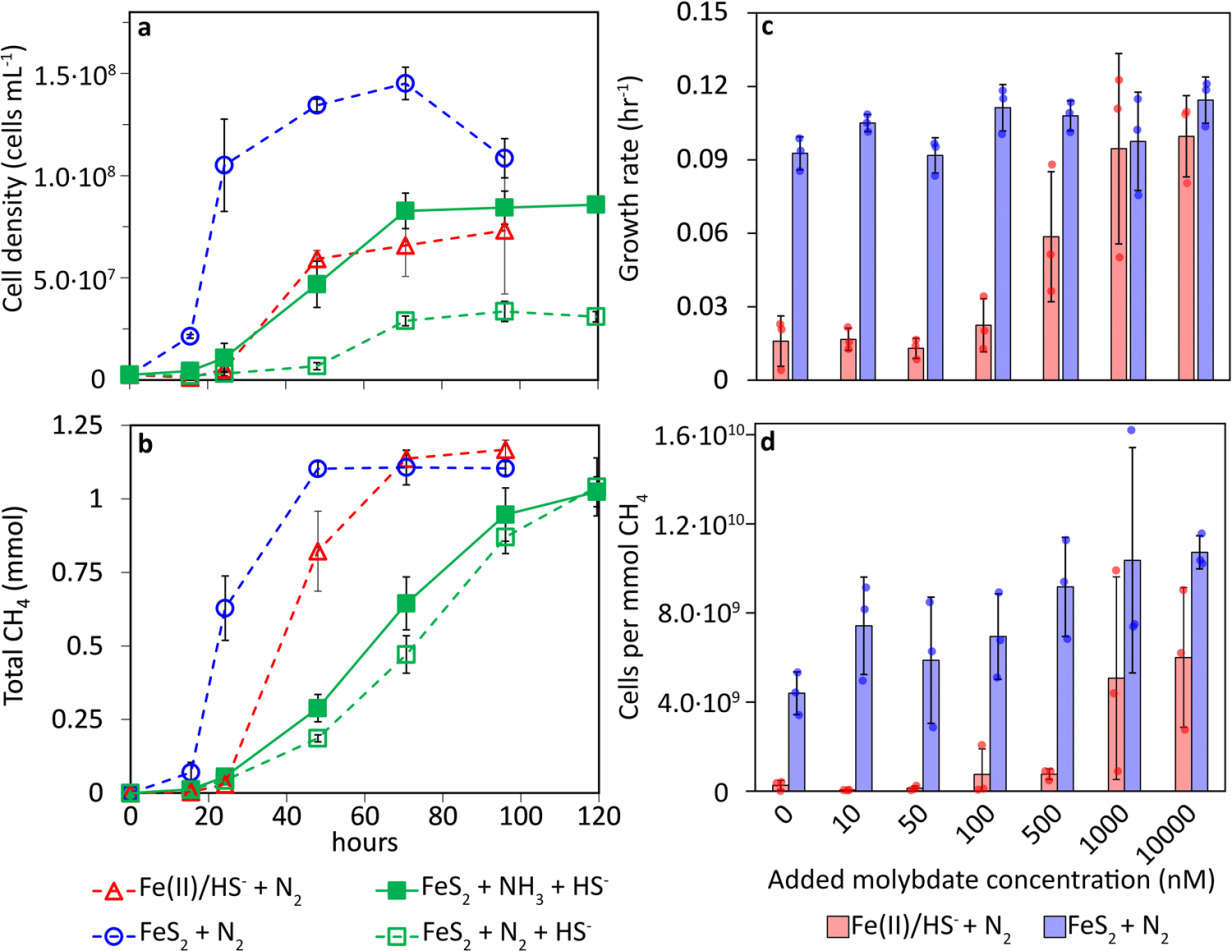
The effect of added sulfide or molybdenum on the growth of *Methanococcus maripaludis* with formate as methanogenesis substrate under nitrogen-fixing conditions with pyrite or ferrous iron and sulfide as the primary iron and sulfur source. Two mM HS^-^ was added to nitrogen-fixing or ammonia-amended cultures provided with pyrite as the primary iron and sulfur source to test its effect on the production of cells (**a**) and methane (**b**). The effect of different molybdate concentrations on the growth rates (**c**) and yields (**d**) of cells grown with either pyrite or ferrous iron and sulfide as the sole iron and sulfur source under nitrogen-fixing conditions are shown. Maximal growth rates (calculated from measured cell densities) were determined over a 5-day period for each replicate. The data presented are the mean and standard deviation of three biological replicates per condition. Ammonia NH_3,_ dinitrogen N_2_, ferrous iron Fe(II), methane CH_4,_ pyrite FeS_2_, sulfide HS^-^.

To further test the hypothesis that euxinic conditions (i.e., excess HS^-^) limit the availability of MoO_4_^2-^ in MmS2 cultures, the growth of N_2_-fixing cells provided with Fe(II)/HS^-^ was compared to N_2_-fixing cells provided with FeS_2_ in the presence or absence of 2 mM added HS^-^. An NH_3_-amended MmS2 culture provided with FeS_2_ and 2 mM added HS^-^ was also tested. As was observed previously, MmS2 grown with FeS_2_ exhibited faster growth and CH_4_ production kinetics and generated higher cell yields than cultures provided with Fe(II)/HS^-^ when fixing N_2_ (Fig. 4a, b). In comparison, FeS_2_-grown cultures with 2 mM added HS^-^, regardless of N source, exhibited significantly slower rates of cell and CH_4_ production, demonstrating that HS^-^ decreases the growth of MmS2 in cells provided with FeS_2_. While both N_2_-fixing and NH_3_-amended cultures provided with FeS_2_ and 2 mM added HS^-^ conditions grew poorly, N_2_-fixing cells were more negatively impacted.

If HS^-^ negatively impacts cellular growth under N_2_-fixing conditions through its effect on MoO_4_^2-^, it would be expected that cells grown on FeS_2_ would have lower requirements for MoO_4_^2-^ in solution than Fe(II)/HS^-^-grown cells since relatively small amounts of HS^-^ are released into solution during FeS_2_ reduction. Previous experiments performed with H_2_-grown, N_2_-fixing MmS2 cells provided with Fe(II)/HS^-^ found that >400 nM MoO_4_^2-^ was required for growth with an optimum of 4000 nM^42^. These values are much higher than those required of other N_2_-fixing organisms. For example, aerobic, N_2_-fixing cyanobacteria such as *Anabaena variabilis*^81^, as well as anaerobic N_2_-fixing purple sulfur bacteria (PSB) inhabiting the interface of oxic/euxinic waters and that serves as a modern analog of the productive continental margins of Proterozoic oceans^82^, can be supported by less than 10 nM MoO_4_^2-^. Interestingly, maximum N_2_ fixation for the PSB was maximal above the chemocline where HS^-^ was near zero^82^. Thus, it seems incongruous that methanogens would require 40-fold more MoO_4_^2-^ than these organisms, despite ancestors of methanogens likely being where Nif originated^6,9^.

To further test this, formate-grown and N_2_-fixing MmS2 cells were provided with a range of MoO_4_^2-^ concentrations with Fe(II)/HS^-^ or FeS_2_ as the sole Fe/S source. Consistent with previous work^42^, MmS2 grown on Fe(II)/HS^-^ showed little to no growth response in conditions with <100 nM MoO_4_^2-^ but growth rates increased substantially when ≥500 nM MoO_4_^2-^ was provided (Fig. 4c**)**. Cell yields did not reach maximum levels in Fe(II)/HS^-^-grown cells until >1000 nM MoO_4_^2-^ was provided (Fig. 4d). Strikingly, cells provided with FeS_2_ grew well under all Mo concentrations tested, including when 0 µM MoO_4_^2-^ was added (Fig. 4c). After this experiment, abiotic reactors containing media and FeS_2_ (no MoO_4_^2-^ added) were analyzed by ICP-MS and were determined to have background (contaminant) Mo ranging from 10 to 30 nM despite using ACS-grade chemicals and acid-washed glassware. Thus, Fe(II)/HS^-^-grown N_2_-fixing cells required >500 nM MoO_4_^2-^ to achieve near-optimal growth rates and yields whereas FeS_2_-grown N_2_-fixing cells required <30 nM MoO_4_^2-^.

Collectively, these results suggest that MmS2 cells grown with FeS_2_ (in the absence of high concentrations of free HS^-^) can access MoO_4_^2-^ at nM concentrations that are >10-fold lower than previously reported for this strain^42^. Further, MmS2 cells can achieve optimal growth rates and yields at MoO_4_^2-^ concentrations that are at levels ~100-fold lower than previously reported^42^. Despite similar growth rates for the FeS_2_ conditions at different MoO_4_^2-^ concentrations, there was a positive trend in the cell yield with increasing MoO_4_^2-^ that indicates higher MoO_4_^2-^ concentrations are still beneficial to these cells (Fig. 4d). These findings are consistent with other studies that have shown N_2_ fixation at low Mo concentrations with cells grown under non-sulfidic conditions^81,82^. Notably, the low levels (10-30 nM) of MoO_4_^2-^ shown to support N_2_ fixation herein are similar to the inferred Mo concentrations of Proterozoic oceans^83–85^). Other studies investigating Mo requirements for other methanogen species fixing N_2_ found 1 to 10 µM MoO_4_^2-^ to be the optimal concentrations^86,87^. There is one other example of methanogens fixing N_2_ at low (<10 nM) MoO_4_^2-^ concentrations; Nishizawa et al. showed that isolates of *Methanocaldococcus* and *Methanothermococcus* from a hydrothermal vent could fix N_2_ with as low as 5 nM or 1 µM MoO_4_^2-^, respectively^59^. This indicates that different methanogen species within the same hydrothermal environment (at different temperatures) can have very different Mo requirements. Importantly, these past experiments were all performed in the presence of >1 mM HS^-^, necessitating a re-evaluation of methanogens’, and other anaerobes’, ability to access MoO_4_^2-^ in the absence of high HS^-^. These data support the hypothesis that N_2_ fixation via Nif is more efficient in low HS^-^ conditions where Mo is more bioavailable.

### Ferruginous conditions favor growth of MmS2 under N_2_-fixing conditions

Excess HS^-^ repressed growth of MmS2 when grown with FeS_2_ (euxinic conditions), regardless of the N source provided (Fig. 4). Next, the growth of N_2_-fixing cells was compared in FeS_2_-grown cultures with medium formulations designed to mimic mildly (100 µM added Fe(II)) and highly (1000 µM added Fe(II)) ferruginous conditions and to mimic mildly (100 µM added HS^-^) and highly (1000 µM added HS^-^) euxinic conditions. Control cultures contained FeS_2_ as the sole source of Fe and S. Cell densities and CH_4_ concentrations were monitored as growth proxies, and HS^-^ was measured as a proxy for FeS_2_ reduction.

**Fig. 5:**
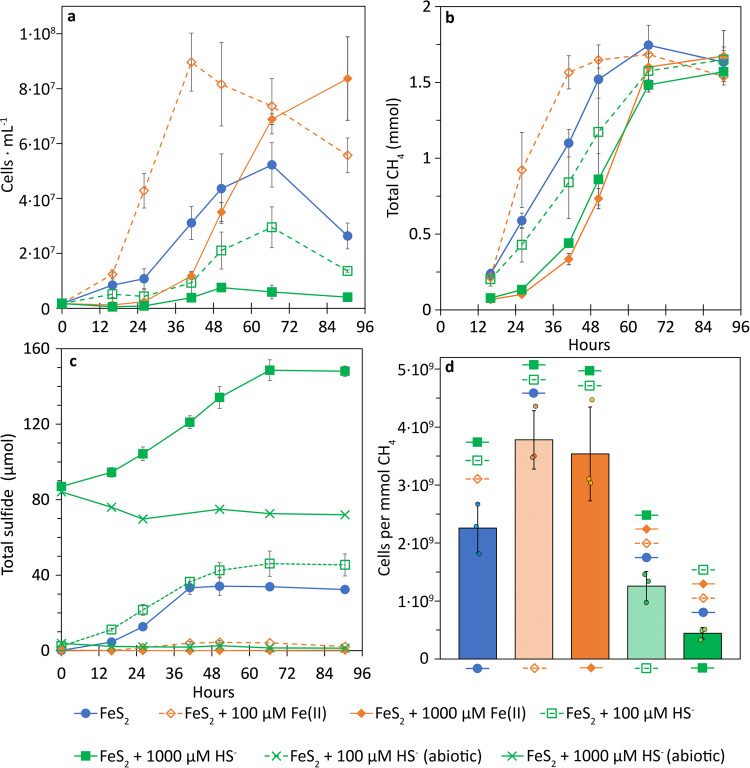
The effect of excess ferrous iron (Fe(II)) or sulfide (HS^-^) on *Methanococcus maripaludis* S2 (MmS2) under nitrogen-fixing conditions. Production of cells (**a**), methane, CH_4_ (**b**), and HS^-^ (**c**) was monitored in cultures of MmS2 provided with 2 mM pyrite (FeS_2_) as the sole Fe/S source, with FeS_2_ and 100 or 1000 µM of Fe(II), or with FeS_2_ and 100 or 1000 µM HS^-^. Cell yields (**d**) in each growth condition were calculated using the initial and maximum cell and CH_4_ concentrations for each replicate. The legend at the bottom of the figure applies to all four panels. Statistically significant (*p* < 0.05, *t*-test) differences were determined pairwise in panel **d** and are depicted above each bar using the symbols from the legend to denote the significantly different pairs. The data presented are the mean and standard deviation of three biological replicates per condition. Ferrous iron Fe(II), methane CH_4_, pyrite FeS_2,_ sulfide HS^-^.

MmS2 cells grew better under the mildly ferruginous condition relative to mildly and highly euxinic conditions, although the highly ferruginous condition caused a longer lag phase (Fig. 5a). Growth of MmS2 cells under euxinic conditions, especially with 1000 µM added HS^-^, was greatly diminished relative to cells grown with FeS_2_ as the control. Despite the differences in the overall densities of cells at the end of the experiment, all conditions produced similar amounts of CH_4_ (Fig. 5b). This led to pronounced differences in final cell yields (Fig. 5d), as well as yields during log phase (Supplementary Fig. 6). In particular, cell yields for the mildly ferruginous condition were the highest even relative to the control condition (FeS_2_ only). Again, yields of cells grown in euxinic conditions were significantly lower than those grown in either ferruginous conditions or in the control (FeS_2_ only) condition. Together, these data suggest that mildly ferruginous conditions are preferred for N_2_-fixing MmS2 cells.

Total sulfide (HS^-^, H_2_S, and acid labile iron-sulfide) was measured in non-filtered culture samples as an indicator of FeS_2_ reduction^41^. Despite all conditions requiring the mobilization of FeS_2_ to access either Fe (euxinic conditions), S (ferruginous conditions), or both Fe and S (control condition), the production of total sulfide differed among conditions (Fig. 5c). For FeS_2_ alone (control conditions), 34 µmol total sulfide was observed by the end of the experiment, while the mildly euxinic condition produced 46 µmol total sulfide. Accounting for the added HS^-^ (100 µM, 7.5 µmol), 38 µmol of total sulfide was produced minus what was assimilated by the cells in mildly euxinic conditions. Further, this indicates a similar amount of cellular FeS_2_ reduction took place in these two growth conditions; however, more FeS_2_ was reduced per cell in the mildly euxinic condition given low cell production. Similarly, in the highly (1000 µM, 75 µmol added HS^-^) euxinic condition, 148 µmol total sulfide was measured at the end of the experiment, indicating that more FeS_2_ was reduced on a per cell basis than control conditions considering low cell production in the former condition (Fig. 5a). Total sulfide production in cultures grown in ferruginous conditions was far less than control cultures (FeS_2_-only). Specifically, mildly (100 µM added Fe(II)) ferruginous conditions produced only 4 µmol total sulfide, while total sulfide remained undetectable in highly (1000 µM added Fe(II)) ferruginous conditions until the final time point where 0.3 µmol was detected. Thus, the amount of FeS_2_ that must be reductively dissolved to meet biosynthetic demands for Fe or S is lower in ferruginous conditions than in euxinic conditions, which may also help to explain better growth under these conditions.

The impact that HS^-^ had on FeS_2_-grown cells is likely attributable to the complex conditions created when HS^-^, FeS_2_, and trace metals (i.e., Mo, Fe(II)) co-occur. High HS^-^ concentrations decrease the thermodynamic favorability of FeS_2_ reduction^41^, which might make FeS_2_ reduction the rate-limiting process in these cultures and possibly explain the slower production of CH_4_ observed in FeS_2_-grown cultures with added HS^-^ (Figs. 4b and  5b). In addition, high HS^-^ may reduce the metal availability of not only MoO_4_^2-^, but also Fe(II) that is released during FeS_2_ reduction. Spietz et al. describe a model for reductive dissolution of FeS_2_ by methanogens wherein FeS_2_ reduction releases HS^-^ into solution and pyrrhotite (Fe_1-x_S) is precipitated as a secondary phase on the mineral surface^41^. Dissolution of Fe_1-x_S results in the release of Fe(II) (but not HS^-^) that can then react with soluble HS^-^ to form FeS_(aq)_ clusters, the presumed source of Fe/S for these cells^39,41^. In abiotic experiments with Fe_1-x_S sequestered in 100 kDa dialysis membranes, it was shown that dissolution and/or diffusion of Fe from Fe_1–*x*_S to the bulk medium was significantly decreased in the presence of 500 µM HS^-^ when compared to no added HS^-^^41^. This may indicate that HS^-^ increases the rate of FeS_(aq)_ nucleation and re-precipitation as mackinawite (FeS_mack_) nanoparticles that are too large to diffuse across the cell membrane. Such particles would be expected to limit Fe (and S) availability to cells^41^. These effects of HS^-^ are presumed to not occur under ferruginous conditions because Fe(II) effectively scrubs HS^-^ or because cells minimize FeS_2_ reduction such that all HS^-^ released from the process is assimilated. It is also important to consider the tendency for trace metals to become adsorbed onto FeS_2_ surfaces that, in turn, reduces their concentration in solution. This is the case for MoS_4_^2-^, the product of complete MoO_4_^2-^ thiolation (see above), which more strongly adsorbs to FeS_2_ surfaces than MoO_4_^2-^^80^.

### Conversion of FeS_2_ to nitrogenase metalloclusters: implications for element cycling in past and contemporary anoxic habitats

Experiments conducted herein were aimed at identifying the most plausible conditions (euxinic or ferruginous) that would have enabled N_2_ fixation via Nif during the early Proterozoic, when phylogenetic data indicate this enzyme evolved^6–8^, or even in the late Archean, when isotopic data suggest this enzyme or its predecessor evolved^21,22^. Experiments were conducted with the marine methanogen, MmS2, which harbors a Nif homolog that belongs to the deepest branching Nif lineage^6,8,9^. Data showed that euxinic conditions (excess of HS^-^ relative to free Fe(II)) negatively affected the growth of N_2_-fixing MmS2 cells, regardless of whether they were grown with Fe(II)/HS^-^ or FeS_2_. This was shown to be due to the effect of HS^-^ on the speciation and availability of Mo. However, under ferruginous conditions, excess Fe(II) titrates HS^-^ leading to its precipitation as FeS_mack_ and FeS_2_ which, in turn, allows Mo to remain bioavailable as MoO_4_^2-^. Indeed, the growth rate and yield of N_2_-fixing MmS2 cells were significantly higher when cells were grown with FeS_2_ and excess Fe(II) (ferruginous conditions) than when grown with *i*) FeS_2_ and excess HS^-^ (euxinic conditions), *ii*) FeS_2_ alone, or iii) under canonical conditions typically used to culture methanogens, excess HS^-^ and Fe(II) (also a euxinic condition).

Collectively, these data indicate that MmS2 prefers FeS_2_ over Fe(II)/HS^-^ during growth with formate as methanogenesis substrate and with N_2_ as the sole N source, conditions that increase the demand of Mo via Fdh, Nif, and Fmd. Additionally, cells grew more efficiently with FeS_2_ when MoO_4_^2-^ was ~100-fold lower in concentration than cells grown with Fe(II)/HS^-^. These data suggest that a ferruginous environment would have favored the function and potentially the origin of Nif on early Earth. In contrast, euxinic conditions pose significant challenges to access thiophilic metals required for N_2_-fixing cells in both ancient and modern environments, thus limiting N_2_ fixation via Nif. Since metal availability is a major driver of the evolution of metalloenzymes (reviewed in^83^), we suggest that Nif likely evolved in an environment that favored the availability of Fe and Mo.

## Methods

### Preparation of minerals

Synthetic FeS_2_ was synthesized in an anaerobic chamber by mixing separate solutions of 16.7 g FeSO_4_ · 7 H_2_O and 17.28 g Na_2_S · 9 H_2_O each dissolved in 50 mL of anoxic, deionized MilliQ H_2_O (MQ) in a 500 mL media bottle. 2.1 g elemental sulfur was then added to the reactor, which was then sealed with a rubber stopper and removed from the anaerobic chamber. The reactor was then incubated for four days at 65 °C followed by another four days at 85 °C. The reactor was then cooled to room temperature and was opened in a chemical fume hood where the resulting mineral was collected into centrifuge tubes and washed several times aerobically with MQ, 1 M HCl, 6 M HCl, and acetone. Finally, the mineral was washed in sterile, anoxic MQ several times within an anaerobic chamber and transferred into a sterile glass serum bottle as a slurry^40^. Dried specimen FeS_2_ was crushed and sieved (63–150 µm particle size) before it was subjected to the same wash series as the synthetic FeS_2_. The specimen FeS_2_ was then dried under a stream of N_2_ and stored in a sterile glass serum bottle until it was weighed in an anaerobic chamber and then added directly to culture bottles. Synthetic FeS_2_ was added to prepared culture bottles as a slurry. Minerals were characterized by X-ray diffraction prior to their use in experiments (Supplementary Fig. 7).

### Strains and media

*M. maripaludis* strain S2 (MmS2), kindly provided by Dr. Kyle Costa, was grown in Fe- and S-free basal medium that contained (g L^−1^): NaCl, 21.98; MgCl_2_ · 6H_2_O, 5.10; NaHCO_3_, 5.00; K_2_HPO_4,_ 0.14; KCl, 0.33; CaCl_2_ · 2H_2_O, 0.10. For NH_3_-amended cultures, NH_4_Cl was added to a final concentration of 0.50 g L^−1^. The basal medium was amended with 25 µM FeCl_2_ · 4H_2_O and 2 mM Na_2_S · 9H_2_O for Fe(II)/HS^-^-grown cells. Sulfide addition experiments tested the effect of 2 mM HS^-^ during growth on FeS_2_. Iron and HS^-^ were added from anoxic, autoclaved stock solutions 30 min. prior to inoculation. For FeS_2_ growth conditions, the basal medium was amended with synthetic FeS_2_ to a final concentration of 2 mM Fe (0.018 g per 75 mL of media) or with 1.5 g of specimen FeS_2_. The higher amount of specimen FeS_2_ was added to account for differences in the estimated surface area of in specimen vs. synthetic FeS_2_^40^. Basal medium was amended (each 1% *v*/*v* final concentration) with a trace element, vitamin, formate, and acetate solutions (Supplementary Table 4). The vitamin solutions were filter-sterilized (0.22 µm) whereas the trace element, formate, and acetate solutions were sterilized individually by autoclaving. All solutions were sparged with filtered (0.22 µm) N_2_. N_2_ and other gases used in this study were passed over a heated column (300 °C) containing H_2_-reduced copper shavings.

To prepare the growth medium, all components, except for NaHCO_3_, were dissolved in MQ H_2_O and then boiled for less than 1 min. After boiling, the bottle containing the medium was removed from the heat and sealed with a butyl rubber stopper with a metal cannula and vent needle and sparged with N_2_ gas for 1 h per liter of medium. After degassing, the bottle was sealed, brought into an anaerobic chamber, and NaHCO_3_ was added after cooling. After adding these components, the pH of the medium was adjusted to 7.00 using anoxic 2 M HCl or 1 M NaOH. Seventy-five milliliters of media were dispensed into 165 mL serum bottles that were then sealed with new and freshly boiled 20 mm blue butyl rubber (Bellco, Vineland, NJ) stoppers and aluminum crimp caps. The headspace of the medium bottles was purged with 80:20 N_2_:CO_2_ for 20 min. The bottles were then autoclaved for 20 min at 123 °C. After autoclaving, FeS_2_, Fe(II), HS^-^, trace elements, vitamins, and organics were added to the concentrations indicated above.

### Cultivation of MmS2

MmS2 was maintained by twice-weekly transfers in a defined medium with Fe(II)/HS^-^, N_2_ gas, and formate. Cultures were inoculated on a 5% *v*/*v* basis with cells grown under N_2_-fixing conditions and that were provided with Fe(II)/HS^-^. All cells were washed in basal, NH_3_-free medium (no additions) by centrifuging at 4696 x *g* for 20 min at 20 °C in a swing-out bucket rotor for all experiments. The density of an aliquot of the washed cells was then immediately enumerated before they were used as inoculum. Cultures were pressurized to 2.87 atm with either 80:20 N_2_:CO_2_ or Ar:CO_2_. All cultures were incubated statically on their sides at 38 °C in the dark.

### Measurement of activity and growth

Headspace gas from microcosms was sampled with an N_2_-flushed syringe and stopcock and diluted with ultra-high purity N_2_ into CaliBond bags (Calibrated Instruments Inc., Manhasset, NY) prior to CH_4_ determination. Liquid samples were taken on the benchtop using an N_2_-flushed syringe and needle. CH_4_ was quantified by gas chromatography and total sulfide was quantified colorimetrically via the methylene blue assay, as described previously^40^. Cells for direct counting were first fixed for four hrs in 2.5% v/v (final concentration) glutaraldehyde at 4 °C and were then counted using a Petroff-Hausser counting chamber on a Nikon YS100 light microscope with a 100x oil objective lens (Nikon, Tokyo, Japan). Fixed cell samples were concentrated by centrifugation at 15,000 x *g* for 15 min and resuspended in the basal medium of a sufficient volume to facilitate cell counting

### Molybdate stability and thiolation experiments

To test the stability of MoO_4_^2-^ in various medium formulations used herein, 75 mL of NH_3_-free base salts medium containing trace elements without MoO_4_^2-^, formate, acetate, or vitamins were added to 165 mL serum bottles. Medium preparation was as described above for cultures of MmS2. The headspace composition was 80:20 N_2_:CO_2_. The medium was amended with Na_2_MoO_4_ · 4H_2_O (10 µM final concentration), Na_2_S · 9H_2_O (2 mM), FeCl_2_· 4H_2_O (25 µM), or synthetic FeS_2_ (2 mM). Reactors were incubated at 38 °C in the dark and were transferred to an anaerobic chamber (97.5% N_2_, 2.5% H_2_) and subsamples for spectroscopy were collected via syringe and needle and placed into disposable plastic cuvettes (1 cm path length) that were sealed with plastic cuvette caps before removing from the anaerobic chamber. The concentration of MoO_4_^2-^ was determined colorimetrically using a modified catechol assay^78^. The assay was modified to increase the concentrations of working solution components four-fold and the sample-to-working solution volume ratio was adjusted to 4:1, with a 1 mL total assay volume that was measured at 400 nm with a Genesys 10 S Vis Spectrophotometer (Thermo Scientific, Waltham, MA). The working solution was prepared and stored in the anaerobic chamber 2-weeks prior to the experiment. For tetrathiomolybdate quantification, 1 mL of the sample was directly measured at 467 nm. UV–Vis scans (300 nm to 525 nm) were performed on a Cary UV–Vis-NIR 6000i spectrophotometer (Agilent Technologies Inc., Santa Clara, CA) using the same samples prepared for tetrathiomolybdate quantification. Sample absorbance values for each assay were compared against a standard curve that was prepared with fresh reagents (Na_2_MoO_4_· 4H_2_O or MoS_4_(NH_4_)_2_).

### Electron microscopy

To characterize the size of cells grown under different conditions, field-emission electron microscopy (FEM) was performed. Five milliliters of cell culture was taken as a subsample from mid-log-phase cultures used in transcriptomics experiments (see below). Cells were fixed for two hrs at room temperature in 2.5% EM-grade glutaraldehyde (Electron Microscopy Sciences, Hatfield, PA) in basal medium. The fixed cells were then filtered onto a previously Au-sputtered 0.2 µm black Isopore polycarbonate filter (MilliporeSigma, Burlington, MA) using gentle vacuum filtration. The cells were then washed by the addition of 10 mL basal medium to the filter. After washing, the cells were subjected to an ethanol dehydration series (by filtration) using molecular grade ethanol (25%, 50%, 70%, 85%, 95%, 100%) and were then stored at 4 °C until FEM was performed at the Imaging and Chemical Analysis Laboratory at Montana State University. Samples were mounted on the FEM holder using double-sided carbon tape and sputtered with a thin film of iridium for conductivity before imaging. FEM was performed using a high-resolution Supra 55VP electron microscope (Zeiss, Thornwood, NY) with a primary electron beam energy of 1 keV at different magnifications. Cell size was determined using ImageJ to manually measure individual cells by recording the length of two normal measurements calibrated to the image scale bar.

### Transcriptomics experiments

Shotgun transcriptomics was performed on cells of MmS2 grown under N_2_-fixing or NH_3_-amended conditions with either Fe(II)/HS^-^ or FeS_2_ as the sole Fe/S source. Fifty milliliters of culture were harvested during the mid-log phase in an anaerobic chamber by centrifugation at 4696 x *g* for 30 min at 4 °C. The supernatant was then discarded, and the tubes were removed from the anaerobic chamber and immediately flash-frozen in liquid N_2_. Cell pellets were stored at −80 °C until RNA extraction.

Total RNA was extracted from cell pellets using TRIzol reagent (Invitrogen, Carlsbad, CA) following the manufacturer’s protocol with minor modifications. One milliliter of TRIzol was added to cell pellets and the resuspended cells were transferred to Lysis E tubes (MP Biomedicals, Irvine, CA) on ice. The cell samples were mechanically lysed by three cycles of 40 sec of bead beating and 5 min of rest at room temperature (~20 °C). After the lysis procedure, 200 µL of molecular grade chloroform was added, the tubes were mixed by inversion and allowed to incubate for 3 min at room temperature. The tubes were then centrifuged for 15 min at 12,000 x *g* at 4 °C and the upper aqueous phase containing RNA was transferred by pipette into a clean 2-mL tube. RNA was precipitated by the addition of 0.5 mL of 100% molecular grade isopropanol that had been pre-chilled to 4 °C followed by 10 min incubation on ice. RNA was then pelleted by centrifugation for 10 min at 12,000 x *g* at 4 °C. The supernatant was removed by pipette, and the RNA was washed in 1 mL of 75 % molecular-grade ethanol. RNA was pelleted by centrifugation for 5 min at 7500 x *g* at 4 °C, the supernatant was removed, and the RNA pellet was air dried for 10 min. Once dried, the RNA was resuspended in 50 µL of RNA-free H_2_O (Fisher Scientific, Waltham, MA) by incubating at 55 °C in a heat block for 10 min. RNA was treated to remove residual DNA by the addition of Turbo DNase (Invitrogen, Waltham, MA) according to the manufacturer’s instructions. The RNA was then subjected to a second round of precipitation, washing, drying, and resuspension steps as described above. RNA was quantified using a Qubit 2.0 fluorometer with a Qubit BR RNA kit (Invitrogen) and the quality was assayed using a NanoDrop ND-1000 spectrometer (Thermo Scientific). DNA contamination was checked by performing PCR amplification using archaeal-specific 16S rRNA primers and ensuring no amplification had occurred by gel electrophoresis. The RNA was then sent to the University of Wisconsin’s Genome Expression Center for quality control, rRNA depletion using custom *Methanococcus*-specific oligonucleotides (Supplementary Table 5), and sequencing on an Illumina NovaSeq 2×150 bp (Illumina, San Diego, CA).

RNA paired-end reads were processed using default settings in TrimGalore! (https://www.bioinformatics.babraham.ac.uk/projects/trim_galore/), a wrapper that implements CutAdapt^88^ to remove adapter sequences and FastQC (https://www.bioinformatics.babraham.ac.uk/projects/fastqc/) to filter reads. Reads were aligned to the reference MmS2 genome (ASM1158v1) using BowTie2^89^. Transcriptional profiles were generated by read counting for each locus using HTSeq^90^, then normalized and analyzed in DESeq2^91^ implemented in R v3.6.0. The RNA-sequencing data reported in this paper have been deposited in the NCBI GEO database (GSE216895).

### Isotope analyses

Duplicate cultures of MmS2 were grown (see above) and pooled to provide sufficient sample mass for isotope analyses of cell biomass and CH_4_. Cultures were grown with Fe(II)/HS^-^ or FeS_2_ under N_2_-fixing or NH_3_-amended conditions and harvested during the late-log phase of growth. Concentrations of CH_4_ were determined from each duplicate and cell densities were determined after the duplicates were combined. Cultures were harvested in an anaerobic chamber after reaching > ~3 × 10^7^ cells per mL by centrifugation in 50 mL centrifuge tubes (Globe Scientific, Mahwah, NJ) for 30 min at 4696 x *g* at 4 °C. The supernatant was removed using a line and needle under low-vacuum and the cells were resuspended in 8 mL of basal medium and transferred to a 15 mL centrifuge tube (Globe Scientific).

To remove bulk FeS_2_ (if present), the sample was underlaid with a Percoll (GE Healthcare, Chicago, IL) working solution with 0.4 M NaCl according to the manufacturer’s instructions using a syringe and cannula. The samples were then spun at 2000 x *g* in a swing-out bucket rotor for 20 min at 4 °C. The overlaying aqueous phase containing cells was removed by pipette and transferred to a clean 50 mL centrifuge tube, to which ~40 mL of fresh basal medium was added and the tubes were mixed by vortex. The tubes were then spun for 30 min at 4696 x *g* at 4 °C to pellet the cells away from any residual Percoll. The supernatant was then removed by vacuum, and the cell pellets were resuspended in 1.5 mL of basal medium and transferred into 2.0 mL screw-top microcentrifuge tubes (Thermo Scientific). A subsample (10 µL) of cells was taken and diluted for cell enumeration after processing. Biomass was concentrated by centrifugation for 20 min at 15,000 x *g* at 4 °C in a fixed-angle rotor, and the supernatant was removed by pipette.

Cell pellets were frozen at −80 °C until shipment to Utah State University Geosciences for C and N stable isotope analyses. Cell pellets were dried aerobically by incubating for 16 h at 60 °C in a drying oven. Once dried, biomass was scraped out of the tubes, weighed, and further prepared for analysis by grinding to a powder and transferring into tin capsules. Isotope analysis of δ^15^N (vs. AIR) and δ^13^C (vs. VPDB) values was then performed by combustion and gas chromatography of the biomass samples at 1000 °C using a Costech 4010 Elemental Analyzer coupled with a Thermo Scientific Delta V Advantage isotope ratio mass spectrometry (GC-IRMS). For δ^13^C analysis of CH_4_, gas samples from cultures used in the above isotope experiments were shipped to Northern Arizona University and analyzed on a Picarro G2201-I cavity ring-down spectrometer. A commercial δ^13^C −60‰ (vs. VPDB) CH_4_ standard (Airgas, Plumsteadville, PA) served as a calibration standard in addition to methane-free air “zero air” for Picarro analyses.

### Statistics and reproducibility

All data shown are mean values of at least three independent replicates, with error bars showing the standard deviation of the replicates. Independent Student’s *t*-tests were used to calculate *p*-values reported herein for all experiments except for transcriptomics experiments, for which *p*-values were determined by a Wald test calculated in the *DEseq2* package implemented in R. Results were considered significantly different when *p* < 0.05.

### Reporting summary

Further information on research design is available in the Nature Portfolio Reporting Summary linked to this article.

## Supplementary information

Supplementary Materials

Description of Additional Supplementary Files

Supplementary Data 1

Reporting Summary

## Data Availability

All data generated or analyzed during this study are included in this published article and its Supplementary Materials. Numerical and replicate data used for all figures are provided in Supplementary Data 1. Sequencing data from transcriptomics experiments are available through the NCBI Gene Expression Omnibus under the accession ID GSE216895. All other data are available from the corresponding author upon reasonable request.
