# Supplementary material for: Influence of sulfide on diazotrophic growth of the methanogen Methanococcus maripaludis and its implications for the origin of nitrogenase
Source: Commun Biol. 2023 Jul 31;6:799. doi: 10.1038/s42003-023-05163-9 (PMC10390477; doi:10.1038/s42003-023-05163-9)
Supplement: Supplementary file 1 — Supplementary Materials [file 42003_2023_5163_MOESM1_ESM.pdf]

Influence of sulfide on diazotrophic growth of the methanogen *Methanococcus maripaludis* and its implications for the origin of nitrogenase

Devon Payne[1], Rachel L. Spietz[1], Dennis L. Newell[2], Paul J. Dijkstra[3], and Eric S. Boyd[1]

SUPPLEMENTARY MATERIALS

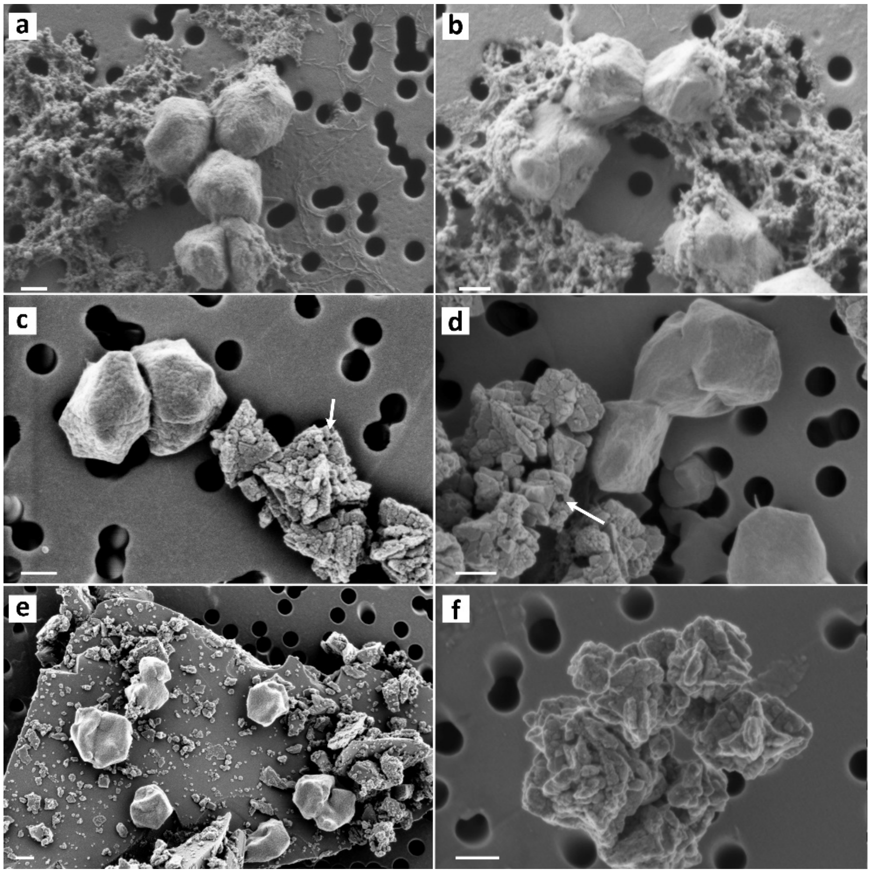

Supplementary Fig. 1. Field-emission scanning electron micrographs of *Methanococcus maripaludis* S2 cells grown under nitrogen-fixing or ammonia-amended conditions with pyrite or ferrous iron and sulfide as the sole provided iron and sulfur source. *M. maripaludis* cells grown with ferrous iron and sulfide with nitrogen (**a**) or with ammonia (**b**) are observed in association with precipitated iron-sulfide minerals. *M. maripaludis* cells grown with synthetic pyrite with nitrogen (**c**) or synthetic pyrite with ammonia (**d**) or with specimen pyrite and nitrogen (**e**) are observed attached to mineral surfaces. Synthetic pyrite from an abiotic reactor with nitrogen is shown (**f**). White arrows added indicate signs of pitting on synthetic pyrite. Scale bar equals 300 nm in all panels.

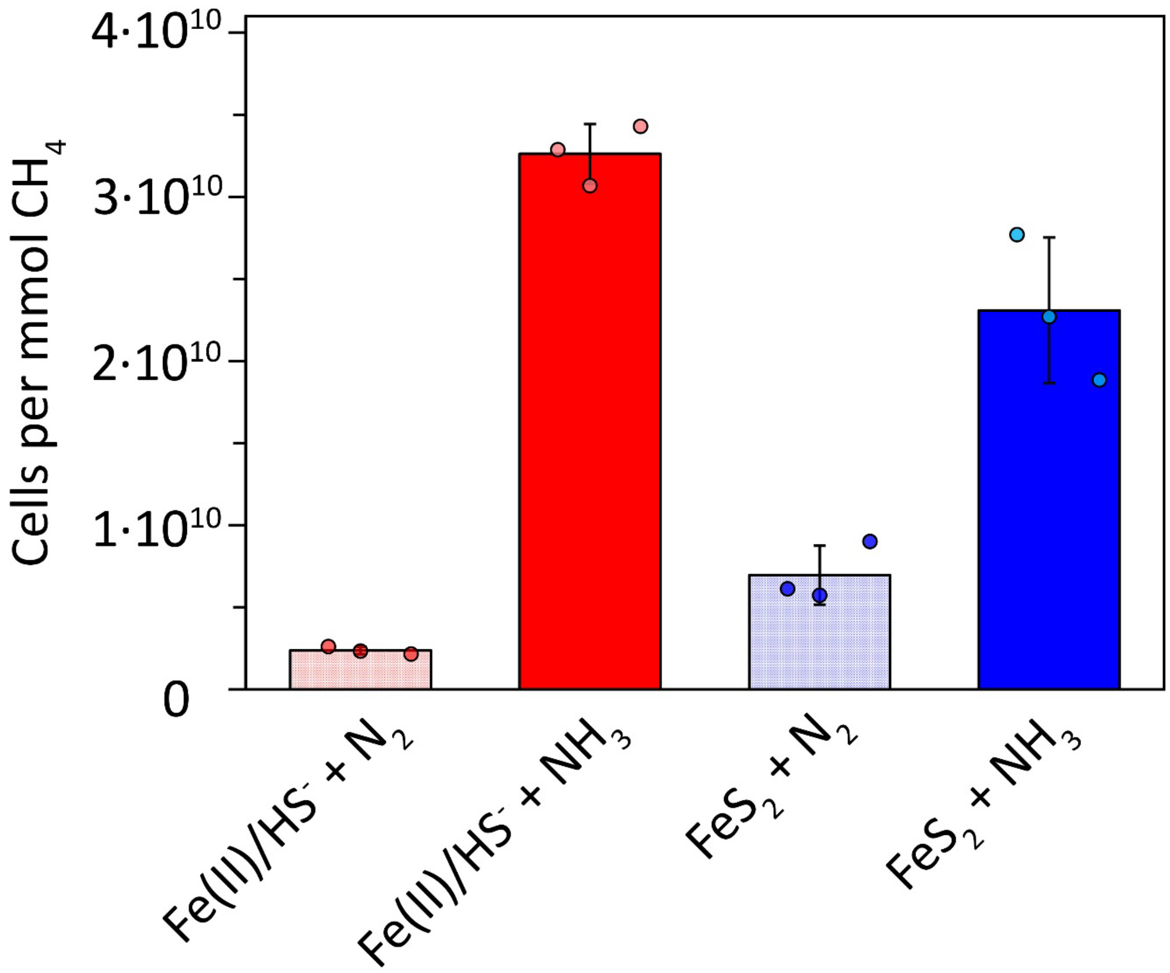

Supplementary Fig. 2. Calculated cell yields of cultures of *Methanococcus maripaludis* S2 grown under nitrogen-fixing or ammonia-amended conditions with pyrite or ferrous iron and sulfide as the sole provided iron and sulfur source. Yield calculations were performed using the production of total methane and cells from the initial timepoint and the timepoint of observed maximal cell density for each condition. Data presented are the mean and standard deviation of three biological replicates per condition. Abbreviations: ammonia, NH_3; dinitrogen, N_2; ferrous iron, Fe(II); methane, CH_4; pyrite, FeS_2; sulfide, HS^- .

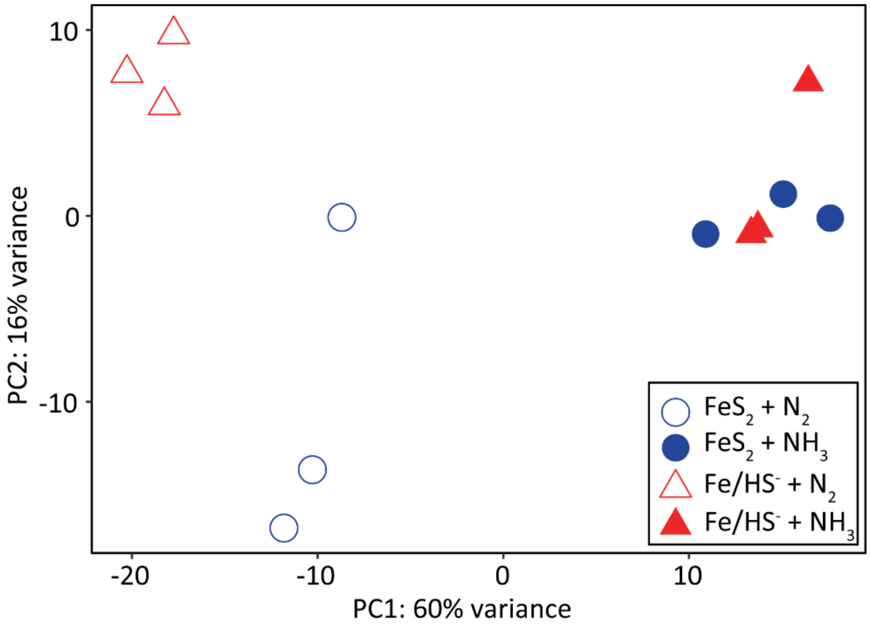

Supplementary Fig. 3. Principal component analysis of differential transcription of genes in *Methanococcus maripaludis* S2 cultures grown under nitrogen-fixing or ammonia-amended conditions with pyrite or ferrous iron and sulfide as the sole provided iron and sulfur source. Cultures were harvested during exponential growth (*see* Supplementary Table 2) for RNA extraction. A total of three replicates were analyzed for each growth condition. Abbreviations: ammonia, NH_3; dinitrogen, N_2; ferrous iron, Fe(II); methane, CH_4; pyrite, FeS_2; sulfide, HS^- .

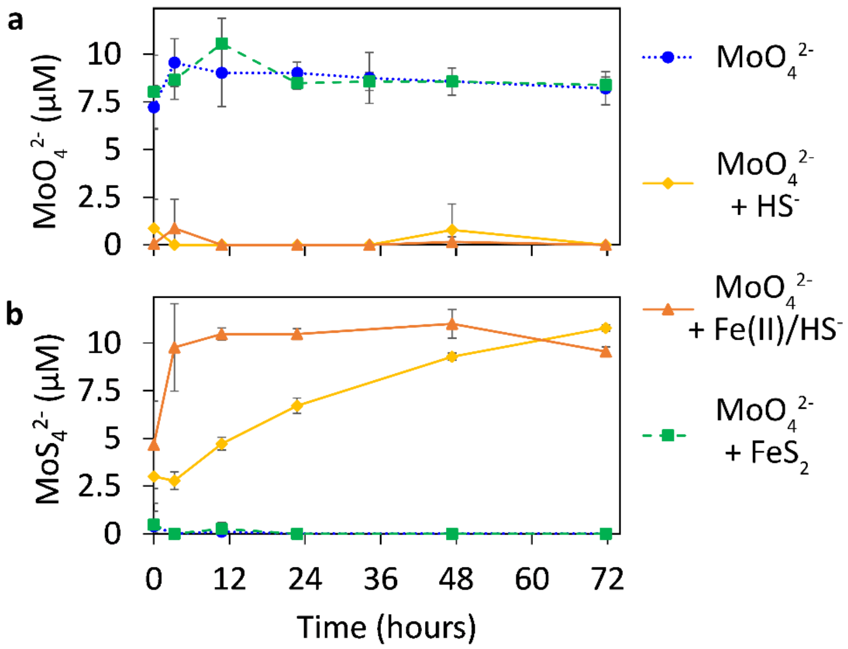

Supplementary Fig. 4. Abiotic transformations of molybdate in the presence of sulfide, ferrous iron, or pyrite. Abiotic experiments containing 10 µM molybdate in basal medium with added trace metals but without ammonia, vitamins, or organic carbon sources were incubated at 38°C. Sulfide, ferrous iron and sulfide, or pyrite were added at the same concentrations as used in growth experiments. The effect of these compounds on molybdate concentrations that were quantified colorimetrically over time using a modified Catechol assay (**a**) and tetrathiomolybdate concentrations via ultraviolet-visible spectroscopy (**b**). The data presented are the mean and standard deviation of three abiotic reactors for each condition. Abbreviations: dinitrogen, N_2; ferrous iron, Fe(II); molybdate, MoO_4^{2-}; pyrite, FeS_2; sulfide, HS^- .

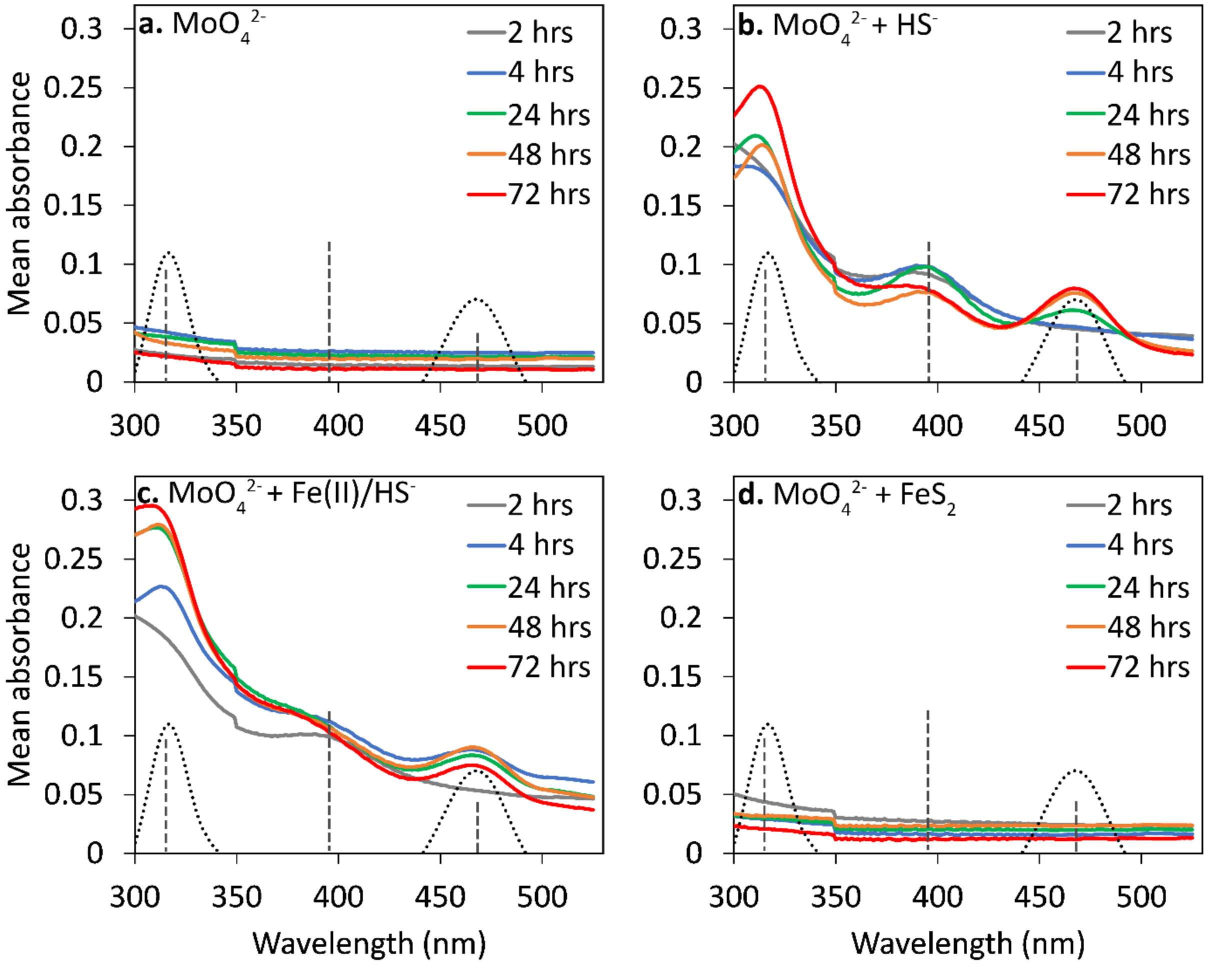

Supplementary Fig. 5. Detection of dissolved thiomolybdate species in abiotic reactors by ultraviolet-visible spectroscopy. Abiotic experiments containing 10 µM molybdate in basal medium with added trace metals but without ammonia, vitamins, or organic carbon sources were incubated at 38°C. No amendments (**a**) or the addition of sulfide (**b**), ferrous iron and sulfide (**c**), or pyrite (**d**) at the same concentrations as were used in growth experiments were tested for their effect on the production of thiomolybdates that are spectroscopically active from 300 to 550 nm. Gray dashed lines represent the general position of intermediate thiomolybdate peaks according to (1). The dotted black line is the spectrum for a 10 µM ammonium tetrathiomolybdate standard in basal medium with no additional amendments. Abbreviations: ferrous iron, Fe(II); molybdate, MoO_4^{2-}; pyrite, FeS_2; sulfide, HS^-.

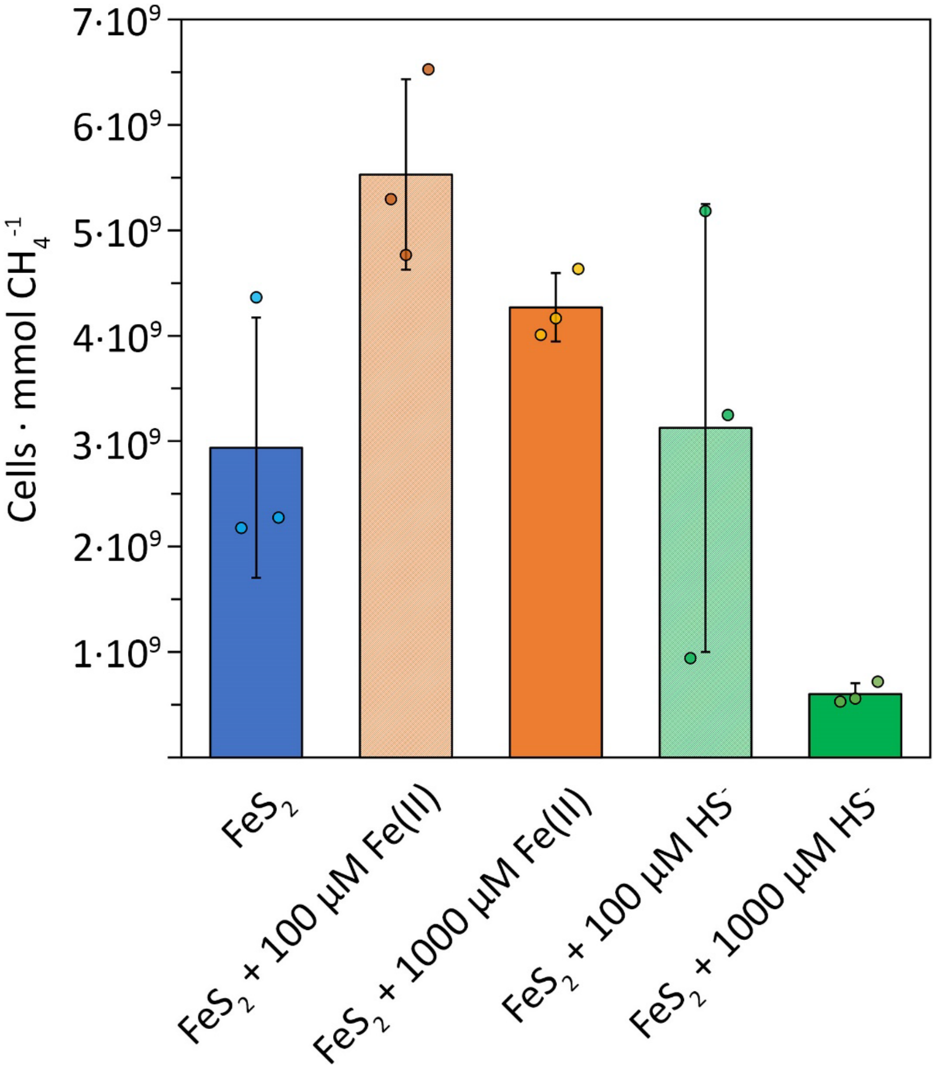

Supplementary Fig. 6. The effect of excess ferrous iron or sulfide on Methanococcus maripaludis log-phase yields under nitrogen-fixing conditions with pyrite and formate. Cell yields were calculated for each condition using measured cell and CH_4 concentrations during exponential growth in cultures provided with 2 mM FeS_2, with FeS_2 and 100 or 1000 μM of Fe(II), or with FeS_2 and 100 or 1000 μM HS^-. Data shown are the mean, standard deviation, and individual replicate data for each condition. Abbreviations: ferrous iron, Fe(II); methane, CH_4; pyrite, FeS_2; sulfide, HS^- .

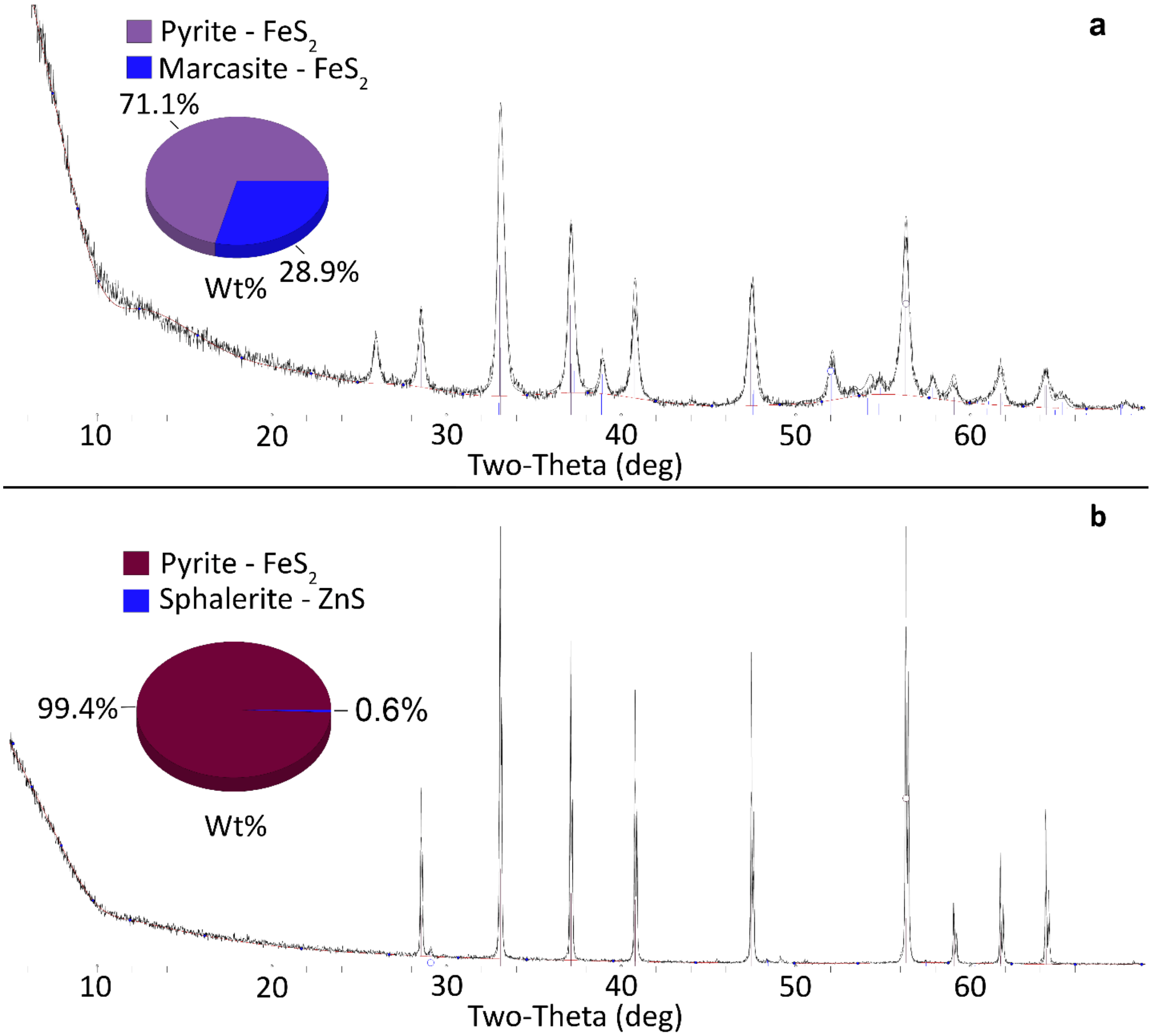

Supplementary Fig. 7. X-ray diffraction spectra from synthetic pyrite (a) and specimen pyrite (b) used in growth experiments. X-ray diffraction spectra were analyzed in Jade using published reference spectra from the Inorganic Crystal Structure Database to identify mineral phases. The calculated weight percent (Wt%) of each identified mineral phase is shown to the left of each spectrum. References for reference spectra for synthetic pyrite (2), marcasite (3), specimen pyrite (4) and sphalerite (5) are provided in the supplementary references.

Supplementary Table 1. Maximum growth rates and cell sizes of nitrogen-fixing or ammonia-amended cells with ferrous iron and sulfide or pyrite

Condition	Growth rate (hr^{-1})			Cell size (μm)			# of cell
	mean		SD	mean		SD	measurements
Fe(II)/HS^- + N_2	0.06	±	0.01	0.75	±	0.16	975
Fe(II)/HS^- + NH_3	0.35	±	0.06	0.89	±	0.19	927
FeS_2 + N_2	0.15	±	0.02	0.67	±	0.13	460
FeS_2 + NH_3	0.27	±	0.02	0.69	±	0.15	923

Abbreviations: ammonia, NH_3; dinitrogen, N_2; ferrous iron, Fe(II); methane, CH_4; pyrite, FeS_2; standard deviation, SD; sulfide, HS^- .

Supplementary Table 2. Cell growth data from nitrogen-fixing and ammonia-amended cells with ferrous iron and sulfide or pyrite as sole iron and sulfur source used in transcriptomics experiments

Condition	Total CH_4 (mmol) Mean ± SD			Total cells Mean ± SD			RNA yield (ng) Mean ± SD			n
$Fe(II)/HS^- + N_2$	0.159	±	0.032	$7.34 \cdot 10^8$	±	$2.10 \cdot 10^8$	351	±	80	3
$Fe(II)/HS^- + NH_3$	0.243	±	0.053	$8.37 \cdot 10^9$	±	$2.53 \cdot 10^9$	13840	±	3154	3
$FeS_2 + N_2$	0.330	±	0.023	$3.71 \cdot 10^9$	±	$7.22 \cdot 10^8$	5186	±	1098	3
$FeS_2 + NH_3$	0.140	±	0.042	$4.31 \cdot 10^9$	±	$2.18 \cdot 10^8$	8899	±	3586	3

Abbreviations: ammonia, NH_3; dinitrogen, N_2; ferrous iron, Fe(II); methane, CH_4; pyrite, FeS_2; standard deviation, SD; sulfide, HS^- .

Supplementary table 3. Additional transcriptomic expression data (VSD) for formylmethanofuran and formate dehydrogenases

Locus tag	Gene	Function	Growth condition expression data							
			Fe(II)/HS^- + N_2		Fe(II)/HS^- + NH_3		FeS_2 + N_2		FeS_2 + NH_3	
			Mean	SD	Mean	SD	Mean	SD	Mean	SD
MMP_RS06400	*fwdH*	Tungsten formylmethanofuran dehydrogenase	14.50	0.13	14.92	0.48	14.45	0.68	14.95	0.13
MMP_RS06405	*fwdF*	Tungsten formylmethanofuran dehydrogenase	14.10	0.07	14.59	0.38	14.11	0.65	14.60	0.12
MMP_RS06410	*fwdG*	Tungsten formylmethanofuran dehydrogenase	8.42	0.53	9.04	0.28	8.51	1.09	9.27	0.03
MMP_RS06415	*fwdD*	Tungsten formylmethanofuran dehydrogenase	12.08	0.01	12.82	0.34	12.14	0.77	12.80	0.04
MMP_RS06420	*fwdA*	Tungsten formylmethanofuran dehydrogenase	14.94	0.05	15.66	0.38	15.18	0.72	15.55	0.01
MMP_RS06425	*fwdC*	Tungsten formylmethanofuran dehydrogenase	13.70	0.09	14.46	0.50	14.07	0.72	14.38	0.02
MMP_RS08715	*fwdB*	Tungsten formylmethanofuran dehydrogenase	16.77	0.09	16.57	0.30	17.02	0.39	16.97	0.12
MMP_RS01110	*fmdE*	Molybdenum formylmethanofuran dehydrogenase	9.27	0.14	9.18	0.18	9.43	0.17	9.12	0.06
MMP_RS02690	*fmdE*	Molybdenum formylmethanofuran dehydrogenase	8.30	0.25	7.45	0.28	8.46	0.99	6.99	0.16
MMP_RS02695	*fmdA*	Molybdenum formylmethanofuran dehydrogenase	10.65	0.15	10.00	0.46	11.02	0.88	9.37	0.12
MMP_RS02700	*fmdC*	Molybdenum formylmethanofuran dehydrogenase	10.45	0.09	9.95	0.42	10.89	0.93	9.32	0.20
MMP_RS02705	*fmdB*	Molybdenum formylmethanofuran dehydrogenase	10.96	0.15	10.61	0.44	11.43	0.79	10.21	0.16
MMP_RS02710	*fmdB*	Molybdenum formylmethanofuran dehydrogenase	11.43	0.18	11.17	0.30	11.54	0.59	10.67	0.08
MMP_RS00795	*fdhA*	Formate dehydrogenase	10.62	0.31	11.22	0.30	10.73	0.43	11.06	0.41
MMP_RS00800	*fdhB*	Formate dehydrogenase	10.58	0.05	11.29	0.32	10.80	0.41	11.22	0.25
MMP_RS06680	*fdhB*	Formate dehydrogenase	16.80	0.29	17.47	0.46	16.91	0.83	16.75	0.05
MMP_RS06705	*fdhC*	Formate transporter	14.60	0.14	15.22	0.42	14.36	1.15	14.81	0.32

Abbreviations: ammonia, NH_3; dinitrogen, N_2; ferrous iron, Fe(II); methane, CH_4; pyrite, FeS_2; standard deviation, SD; sulfide, HS^- .

Supplementary Table 4. Composition of stock solutions added to basal media for growth experiments.

Compound	g/L
Trace elements solution	
Nitriloacetic acid	1.500
$MnCl \cdot 4H_2O$	0.085
$CoCl_2 \cdot H_2O$	0.100
$ZnCl_2$	0.047
$NiCl_2 \cdot 6H_2O$	0.025
Na_2SeO_3	0.200
$Na_2MoO_4 \cdot 2H_2O$	0.100
$Na_2WO_4 \cdot 2H_2O$	0.100
$CuCl_2 \cdot 2H_2O$	0.068
Vitamin solution	
Pyridoxine HCl	0.0100
Thiamine HCl	0.0050
Riboflavin	0.0050
Nicotinic acid	0.0050
Ca D(+) pantothenate	0.0050
Biotin	0.0020
Folic acid	0.0020
Vitamin B12	0.0001
Formate solution	
Sodium formate	400.00
Acetate solution	
Sodium acetate $\cdot 3H_2O$	136.08

Supplementary Table 5. Sequences of oligo probes uses in transcriptomics experiments.

Oligo probe	Sequence
Supplemental Probe Pool_1	CCTCGCGGTACGAGCTGACGATGGCCATGCACCACCTCTCAGCGCTTCAG
Supplemental Probe Pool_2	GGAAGTTGATGCCTCCCAAATTCCCGCGCGATATCCAACGCACGGTACTC
Supplemental Probe Pool_3	CGCTACCCCTGAAGTACCCTTGTCCTCTCCCGGTCCCAAGTCCAGCAGTA
Supplemental Probe Pool_4	ATGCGGACCTATCGTTGCCCGCTCCTTCCTCTGACTTAGCGCCAGCGGTC
Supplemental Probe Pool_5	CTCACCTAGGGGCACCAGTGTCGGTTCTGGGTACGGACATCTAAAATCCT
Supplemental Probe Pool_6	CTGAATCGATTACGCTTACGGGACTTTCACCCTCTATAGTGTGACGTTCC
Supplemental Probe Pool_7	CCCGAAGTTACGGAGCCAATTTGCCGACTTCCCTCGACTAGATTCATCCG
Supplemental Probe Pool_8	CCCAAGTTCGACCCTAGCCAAAGGCCAAGGCCTCACTTCAACCCAACTCG
Supplemental Probe Pool_9	CGGCGAACTTAACGGCTTCCCTTCGGCACTAGATGGGCACGGGGCCCACC
Supplemental Probe Pool_10	CCGGGCTCGATTGGCCTTTCACCCCTAGACCGAGGTCAGAAGAGTGCTTC
Supplemental Probe Pool_11	GTACACTCACAACCATGCCCCAACGACAGGCTGCAGTAAAGCTCCACGGG
Supplemental Probe Pool_12	AACAGCGGAGTCCTGACCCGCTTGAGCCAACCTTTGGGCCCTCCTGATGC
Supplemental Probe Pool_13	GGTGGTGTCCCAGGGTCGCCTCCACCTTTCCTGGCGAAAAGGTTTCGATG
Supplemental Probe Pool_14	TTCCTCAAGCTGTTTACACTTGATAAAAGCCCATGCTGTGCATGAGCACT
Supplemental Probe Pool_15	GCTTCACGTGGGCGAGTTACAGCCCACGATCCGAACTACGACTAGGTTTA
Supplemental Probe Pool_16	TGCAGGCCTCCATCCCTCAGTTGAGGGACTTCACCTTGCCCCGGCCTAGA
Supplemental Probe Pool_17	GATCTTCATTTCTACGAGGTCCACTCGACTTCACAGCCGAACTTCTGCCC
Supplemental Probe Pool_18	CCCTACGCCTACGATCGTGAAGATCTTAAGCTCGCCACAACAATATACTC
Supplemental Probe Pool_19	CCACCCGTTGTGGTGCTCCCCCGCCAATTCCTTTAAGTTTCAGTCTTGCG
Supplemental Probe Pool_20	CTGTGGAACCCTTGCCCCTTCGGCGGTGGGGATTCTCACCCCACTTATGC
Supplemental Probe Pool_21	TTAACCACGTGTTAATGAGCCGTCCGCCATGGCCGAAACCATAGACTCGC
Supplemental Probe Pool_22	ATCCGCGTTCAATCGCCTCTAATAACGGAATCTCTATTGATTTCTCTTCC
Supplemental Probe Pool_23	GGTGTGTGCAAGGAGCAGGGACACATTCACCGCAGTATTATGAACTGCGA
Supplemental Probe Pool_24	GTTATTACAACTCTCCCCGGACTTATAAGCTTGAATGCCCAGACAAGAAC
Supplemental Probe Pool_25	ATCCCCGGGGTAGCTTTTTTGTCATCCCTGGCCCCCATCAGTGAGGCACA
Supplemental Probe Pool_26	CAGGTTCAATGACTGTATGCGTCTCGCCTGAGCTTATCGCAGCTTACCAC
Supplemental Probe Pool_27	TAGTTGCCTTTGGGGCCGTGTCATACCATACCTATCTGGTTTCAGGCTCT
Supplemental Probe Pool_28	CGGCTTTCGCCTCTTCGTTGCGTTTTGTTGGCAACAAAGTCAGGCTGGCT
Supplemental Probe Pool_29	TGACCGTCATCATGCTGTCGCTCCTGGTGAGATGCCCGGCGTTGAATCCA
Supplemental Probe Pool_30	GCACTTTCGTGCATTGCGGAGGTTTCGCGCCTGCTGCGCCCCGTAGGGCC
Supplemental Probe Pool_31	GCTCATGATCCCCTTTAATGGGCGAACAGCCCCACCCTTGGGTCCTGCTG
Supplemental Probe Pool_32	CAAGTCTCGGTTATTCCCGTCTCGGACATACAGCTTACCCGTATGCCCTC
Supplemental Probe Pool_33	GCTTCAATCCCCCGCGTTCCCAATCCTTAACGGATTACAAAGTAGGAAGT
Supplemental Probe Pool_34	CGACGACAAGTTCGGAGTTTGACAAAACAGCGAGGAATTTCTTCCCCTAA
Supplemental Probe Pool_35	CCCTTTCCTCTCAAAGAAGTTAACTCGAGAGTTAGGACCGGCTTACCCAC
Supplemental Probe Pool_36	GGGCAGGCGTCGGCCTTAGTACACACCTTTTCAGGCTCGCTAAGACCTAT
Supplemental Probe Pool_37	TGGAAGTCTCCGGCCTTTGCGCCGGAATGGTAGGTTCACCGGATTCTAGT
Supplemental Probe Pool_38	AAAAACCGACATCGAGGTAGCAAGCCGCGGGGTCGATATGGGCTCTTGCC
Supplemental Probe Pool_39	CGTCGGACCCGTTCCAGACAAGTGCCTTCGCCATAGGTGGTCCTTCAAGG
Supplemental Probe Pool_40	CAGACTGCTTTTTCTATGGGGTATTATCCTCAGTTTCCCAAGGTTATCCC
Supplemental Probe Pool_41	AGGAGGTGATCCAGCCGCAGGTTCCCCTACGGCTACCTTGTTACGACTTC
Supplemental Probe Pool_42	AAGTGGCCACCACTCGGGCCGTCGGTGTTACCGCGGCTGCTGGCACCGAA
Supplemental Probe Pool_43	CACCAACTACCTAATCGAGCGCAGTCCAATCCTCAGGCGCATATGCTTTC
Supplemental Probe Pool_44	AAGGTGCTTTTCAGCTTTCCCTCGCGGTACTAGTACACTATCGGTCTTGG
Supplemental Probe Pool_45	CGGTTAAGCCGCAGGATTTAAACAAGGACTTACTGGACCGGCTACGGACG
Supplemental Probe Pool_46	TATAGTTACCCCCGCCGTTTACTGGCGCTTCGCCCGGTTGGAACCGGGGT
Supplemental Probe Pool_47	GAAACGGACGACACAACCCTGGCTCTATACTTCGTACAGCCGTCTTGCGA
Supplemental Probe Pool_48	CCTGCTGTCTTAGACCAAGGACGCCCTTCGTGTTCACACTTAATCGGCAC
Supplemental Probe Pool_49	TCTCGTTACGCCATTCATGCAGGTCGGAACTTGCCCGACAAGGAATTTCG
Supplemental Probe Pool_50	CACGGGCTCCAGAGATCGGCCGAACCCTCCTAACGGAGAGCTCATCACTC
Supplemental Probe Pool_51	TTTTCAGGGTTGCGGCCCATTGTCCTAGCCATTGTAGCCCGCGTGTAGCC
Supplemental Probe Pool_52	AAAGTTTACGGCCAGGACTACCCGGGTATCTAATCCGGTTCGCGCCCCTG
Supplemental Probe Pool_53	CTACAGGATCACCTTTCGGTGCCCTTGGGTCTCGGCGGCCGATTTAGCCC
Supplemental Probe Pool_54	TCGGACCTCCCTGGTCACTGCGACCAGCGATCCCATATATAAACAAGATC
Supplemental Probe Pool_55	GTCCCCATCTCCGGGCTTCCTCTCTCAAGGCCCGTACCGATCGTAGGCTT
Supplemental Probe Pool_56	TCTCCCGGAGGAAAAAGTAGCAACATAGGGCACGGGTCTCGCTCGTTACC
Supplemental Probe Pool_57	CCAATTTATCTCTTGAACTATTAGTACTAGCGGGCTGAACATCTCGGAAA
Supplemental Probe Pool_58	CCGCCCCAGCCAAACTGCCCACCTACCGGTGTCCCCTAAAAGGGTAAGGA
Supplemental Probe Pool_59	AAGCGTCGGAAACTAACCTAGTGTTGCCACGCGTATTTAGATGGCACAGG
Supplemental Probe Pool_60	TCATATTGCTGTGACTCCGGGCCCTTAAGACCCCGTCCCTCAAGATTTTG

Supplemental Probe Pool_61	TCAGGATGAATTTCGAGCTTAGATGCTTTCAGCTCTTATCTCTTGGCGCG
Supplemental Probe Pool_62	CCCTTGATCTCGACAGGTGAGCTGTTACGCACTCGTTAAAGGATGGCTGC
Supplemental Probe Pool_63	GCCTTGTCAGACAACCGATCGACTAGAGGCGCCGACGGCCCGTTCCTCTC
Supplemental Probe Pool_64	TTCCCTCAGACAGCCAACACATCCAGCAGATAGCAACCAACCTGTCTCAC
Supplemental Probe Pool_65	CCCCTAGCCTATCAAACCTGTCTTCTACAGATGTTCTCGTTCCCGAAGGA
Supplemental Probe Pool_66	TCTACCTTGCCGACTATCTAAACTCCGGCTGACCTGAGAGTCACTTCGGA
Supplemental Probe Pool_67	TTCTCAACAGGCGAAAAGAAGGCCCTATAACACCACATCTCCCCGAAGGG

SUPPLEMENTARY REFERENCES

1. B. E. Erickson, G. R. Helz, Molybdenum(VI) speciation in sulfidic waters:: Stability and lability of thiomolybdates. *Geochimica et Cosmochimica Acta* **64**, 1149-1158 (2000).
2. M. Rieder *et al.*, Arsenic in iron disulfides in a brown coal from the North Bohemian Basin, Czech Republic. *International Journal of Coal Geology* **71**, 115-121 (2007).
3. T. Chattopadhyay, H. G. von Schnering, High pressure X-ray diffraction study on p-FeS_2, m-FeS_2 and MnS_2 to 340 kbar: A possible high spin-low spin transition in MnS_2. *Journal of Physics and Chemistry of Solids* **46**, 113-116 (1985).
4. P. Bayliss, Crystal structure refinement of a weakly anisotropic pyrite. *American Mineralogist* **62**, 1168-1172 (1977).
5. B. K. Agrawal, P. S. Yadav, S. Agrawal, Ab initio calculation of the electronic, structural, and dynamical properties of Zn-based semiconductors. *Physical Review B* **50**, 14881-14887 (1994).
