# Supplementary material for: Influence of sulfide on diazotrophic growth of the methanogen Methanococcus maripaludis and its implications for the origin of nitrogenase
Source: Commun Biol. 2023 Jul 31;6:799. doi: 10.1038/s42003-023-05163-9 (PMC10390477; doi:10.1038/s42003-023-05163-9)
Supplement: Supplementary file 4 — Reporting Summary [file 42003_2023_5163_MOESM4_ESM.pdf]

nature portfolio

Corresponding author(s): COMMSBIO-22-4123-T
Eric S Boyd

Last updated by author(s): Jul 19, 2023

Reporting Summary

Nature Portfolio wishes to improve the reproducibility of the work that we publish. This form provides structure for consistency and transparency in reporting. For further information on Nature Portfolio policies, see our Editorial Policies and the Editorial Policy Checklist.

Statistics

For all statistical analyses, confirm that the following items are present in the figure legend, table legend, main text, or Methods section.

n/a	Confirmed	
☐	☒	The exact sample size (*n*) for each experimental group/condition, given as a discrete number and unit of measurement
☐	☒	A statement on whether measurements were taken from distinct samples or whether the same sample was measured repeatedly
☐	☒	The statistical test(s) used AND whether they are one- or two-sided<br>*Only common tests should be described solely by name; describe more complex techniques in the Methods section.*
☐	☒	A description of all covariates tested
☐	☒	A description of any assumptions or corrections, such as tests of normality and adjustment for multiple comparisons
☐	☒	A full description of the statistical parameters including central tendency (e.g. means) or other basic estimates (e.g. regression coefficient) AND variation (e.g. standard deviation) or associated estimates of uncertainty (e.g. confidence intervals)
☐	☒	For null hypothesis testing, the test statistic (e.g. *F*, *t*, *r*) with confidence intervals, effect sizes, degrees of freedom and *P* value noted<br>*Give P values as exact values whenever suitable.*
☒	☐	For Bayesian analysis, information on the choice of priors and Markov chain Monte Carlo settings
☒	☐	For hierarchical and complex designs, identification of the appropriate level for tests and full reporting of outcomes
☐	☒	Estimates of effect sizes (e.g. Cohen's *d*, Pearson's *r*), indicating how they were calculated

Our web collection on statistics for biologists contains articles on many of the points above.

Software and code

Policy information about availability of computer code

Data collection	No software was used
Data analysis	TrimGalore!, CutAdapt, FastQC, BowTie2, HTSeq, DESeq2, R v3.6.0

For manuscripts utilizing custom algorithms or software that are central to the research but not yet described in published literature, software must be made available to editors and reviewers. We strongly encourage code deposition in a community repository (e.g. GitHub). See the Nature Portfolio guidelines for submitting code & software for further information.

Data

Policy information about availability of data

All manuscripts must include a data availability statement. This statement should provide the following information, where applicable:

- Accession codes, unique identifiers, or web links for publicly available datasets
- A description of any restrictions on data availability
- For clinical datasets or third party data, please ensure that the statement adheres to our policy

All data generated or analyzed during this study are included in this published article and its Supplementary Materials

March 2021

Human research participants

Policy information about studies involving human research participants and Sex and Gender in Research.

Reporting on sex and gender	N/A
Population characteristics	N/A
Recruitment	N/A
Ethics oversight	N/A

Note that full information on the approval of the study protocol must also be provided in the manuscript.

Field-specific reporting

Please select the one below that is the best fit for your research. If you are not sure, read the appropriate sections before making your selection.

☐ Life sciences ☐ Behavioural & social sciences ☒ Ecological, evolutionary & environmental sciences

For a reference copy of the document with all sections, see nature.com/documents/nr-reporting-summary-flat.pdf

Ecological, evolutionary & environmental sciences study design

All studies must disclose on these points even when the disclosure is negative.

Study description	We sought to examine the effect of excess sulfide or ferrous iron on N2 fixing methanogen cells. Since these cells are where nitrogenase first originated, the data will provide insight into potential conditions that favored the origin of this critical enzyme. In particular we were keen to examine this question because of the numerous studies that have suggested that sulfide negatively impacts the availability of molybdenum, an element that is required for molybdenum-dependent nitrogenase (Nif).
Research sample	We worked with a model methanogen, Methanococcus maripaludis S2, since it encodes a single nitrogenase homolog (Nif) that belongs to the earliest branching Nif lineage. Further, the strain can grow with formate and this will place additional molybdenum demands on cells since this enzyme is molybdenum dependent.
Sampling strategy	This experiment utilized a combination of growth assays, geochemical assays, isotopic assays, and molecular assays. Pilot experiments were conducted to identify how frequently to sample (without over sampling and significantly disturbing cultures) and these sampling designs were used throughout the experiments.
Data collection	Most of the data was recorded by Dr. Payne in laboratory notebooks that were then transferred to excel datasheets. Molecular (transcriptomic data) was collected via shotgun sequencing of RNA and this was done on triplicate samples sent tot he University of Wisconsin where their staff processed the samples.
Timing and spatial scale	These are described and shown individually for each experiment in figures 1, 4, and 5. Details of when samples were collected for data presented in figures 2 and 3 are provided in the text
Data exclusions	No data were excluded
Reproducibility	All experimental data reported here are derived from triplicate experiments
Randomization	This is not relevant to our study as we focused on a single organism.
Blinding	Both Drs. Payne and Spietz collected data independent of each other and these were then analyzed together and this led to the same interpretation.

Did the study involve field work? ☐ Yes ☒ No

Reporting for specific materials, systems and methods

We require information from authors about some types of materials, experimental systems and methods used in many studies. Here, indicate whether each material, system or method listed is relevant to your study. If you are not sure if a list item applies to your research, read the appropriate section before selecting a response.

Materials & experimental systems

n/a	Involved in the study
☒	☐ Antibodies
☒	☐ Eukaryotic cell lines
☒	☐ Palaeontology and archaeology
☒	☐ Animals and other organisms
☒	☐ Clinical data
☒	☐ Dual use research of concern

Methods

n/a	Involved in the study
☒	☐ ChIP-seq
☒	☐ Flow cytometry
☒	☐ MRI-based neuroimaging
